# Pharmacokinetics of a single 1g dose of azithromycin in rectal tissue in men

**DOI:** 10.1371/journal.pone.0174372

**Published:** 2017-03-28

**Authors:** Fabian Y. S. Kong, Thusitha W. Rupasinghe, Julie A. Simpson, Lenka A. Vodstrcil, Christopher K. Fairley, Malcolm J. McConville, Jane S. Hocking

**Affiliations:** 1 Centre for Epidemiology and Biostatistics, Melbourne School of Population and Global Health, University of Melbourne, Melbourne, Australia; 2 Metabolomics Australia, School of BioSciences, University of Melbourne, Melbourne, Australia; 3 Monash University Central Clinical School and Melbourne Sexual Health Centre, Melbourne, Australia; 4 Metabolomics Australia, Bio21 Institute for Molecular Science and Biotechnology, University of Melbourne, Melbourne, Australia; Imperial College London, UNITED KINGDOM

## Abstract

Chlamydia is the most common bacterial sexually transmitted infection among men who have sex with men. Repeat infection following treatment with 1g azithromycin is common and treatment failure of up to 22% has been reported. This study measured the pharmacokinetics of azithromycin in rectal tissue in men following a single 1g dose to assess whether azithromycin reaches the rectal site in adequate concentrations to kill chlamydia. Ten healthy men took a single oral dose of 1g azithromycin and provided nine self-collected swabs and one blood sample over 14 days. Participant demographics, medications, sexual behaviour, treatment side effects, lubricant use and douching practices were recorded with each swab. Drug concentration over time was determined using liquid chromatography–mass spectrometry and total exposure (AUC_0-∞_) was estimated from the concentration-time profiles. Following 1g of azithromycin, rectal concentrations peaked after a median of 24 hours (median 133mcg/g) and remained above the minimum inhibitory concentration for chlamydia (0.125mcg/mL) for at least 14 days in all men. AUC_0-∞_ was the highest ever reported in human tissue (13103((mcg/g).hr)). Tissue concentrations were not associated with weight (mg/kg), but data suggest that increased gastric pH could increase azithromycin levels and diarrhoea or use of water-based lubricants could decrease concentrations. High and sustained concentrations of azithromycin were found in rectal tissue following a single 1g dose suggesting that inadequate concentrations are unlikely to cause treatment failure. Factors effecting absorption (pH and diarrhoea) or drug depletion (douching and water-based lubricants) may be more important determinants of concentrations *in situ*.

## Introduction

*Chlamydia trachomatis* (CT) is the most common bacterial sexually transmitted infection (STI) worldwide. [1] In Australia, an estimated 40% of diagnoses are among men, [2] and among men who have sex with men (MSM), the prevalence of rectal chlamydia is about 6%. [3]

Current guidelines from the Centre for Disease Control and Prevention recommend that MSM with uncomplicated chlamydia infections be treated with either a single 1g of azithromycin or seven days (100mg twice daily) of doxycycline. [4] However, with repeat rectal chlamydia rates of up to 22% reported following treatment with 1g azithromycin, [5] there is increasing concern about azithromycin treatment failure to the extent that both the European [6] and Australian [7] guidelines now recommend rectal infections be treated with seven days of doxycycline as the first line treatment.

A recent meta-analysis comparing the efficacy of 1g azithromycin and seven days of doxycycline for treating rectal chlamydia estimated an efficacy of 82.9% for azithromycin and 99.6% for doxycycline. [5] However, these efficacy estimates were based on observational data only as there have been no RCTs conducted to date comparing the efficacy of these two treatments for rectal chlamydia. Until an RCT is undertaken, the efficacy of azithromycin for rectal chlamydia infection remains uncertain. [8, 9] While pharmacokinetic data demonstrate that azithromycin reaches urogenital, [10] gynecological [11, 12] and gastric tissue [13, 14] (a proxy for rectal tissue) in adequate concentrations to kill infections such as chlamydia, no such data are available for rectal tissue.

Azithromycin is a broad spectrum, macrolide antibiotic with bacteriostatic activity against susceptible bacteria. While the oral absorption of azithromycin remains low (absolute bioavailability of 37%), it is rapidly absorbed with peak blood concentrations occurring at 2–3 hours post dose. [15] Following absorption, it is transported to the site of infection via phagocytic cells released during the host immune response to infection. [16] Azithromycin penetrates most human tissue, has a long serum half-life of 68 hours and is predominantly excreted in faeces as a result of its elimination in bile [17] and incomplete gastrointestinal tract absorption. [18] It is possible that external factors such as pre-sex douching [19] and water-based lubricants [20] could disrupt the mucus membranes of the rectal tissue and alter azithromycin tissue concentrations *in situ*. Additionally factors that can increase the environmental pH of azithromycin (e.g. gastric acid lowering drugs) could also increase azithromycin’s efficacy [21] due to possible increases in intracellular penetration of the drug. [22]

This study aimed to measure the concentration of azithromycin in self-collected rectal swabs and use these data to make inferences about the pharmacokinetics of azithromycin in rectal tissue in men following a single dose of 1g.

## Methods

### Recruitment of participants

**Fig 1:**
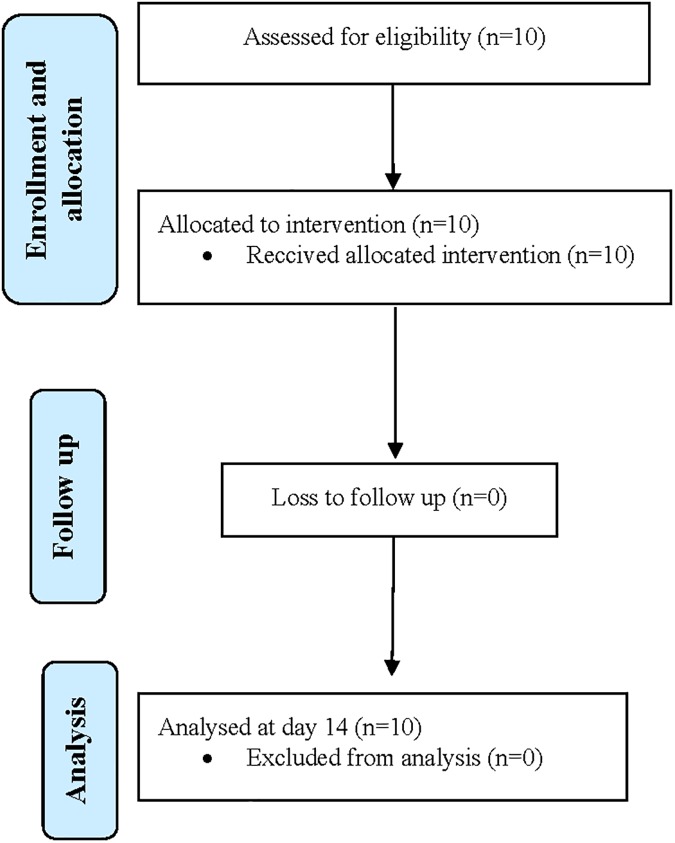
Flow chart indicating different phases of the study. Men were eligible if they were HIV/STI free, aged ≥18 years, and had adequate English and comprehension to provide written informed consent. Exclusion criteria were any antibiotic use in the previous two weeks, commercial sex work and any current drug use likely to interact or be contraindicated to azithromycin use. Recruitment and intervention took place in a large metropolitan sexual health centre where they were offered a standard STI screen and reimbursed $100 for their time and transport.

Study participants were recruited and followed up between 1 January 2016 to 30 May 2016 through advertising on a University of Melbourne staff email list and word of mouth (Fig 1).

Following recruitment, men completed a questionnaire, collected a self-collected rectal swab and were given the prescribed treatment of a single 1g dose of azithromycin by the research nurse under direct observation. The drug was taken with food. Men were then followed up for 14 days. Self-collected rectal swabs were used as a surrogate measure of azithromycin concentration in the rectum, herewith referred to as rectal tissue.

The study was prospectively registered with the Australian drug regulatory authority, the Therapeutic Goods Administration (TGA), Clinical Trial Notification (CT-2015-CTN-03237-1 v2) in compliance with the requirements of the Human Ethics Committee and retrospectively with the Australian New Zealand Clinical Trials Registry (Trial ID: ACTRN12616001666415). The authors confirm that all ongoing and related trials for this drug/intervention are registered.

There were no protocol deviations. There was only one treatment group.

### Participant data

Men’s age, weight and concurrent medications were recorded at recruitment. During the follow up study period, participants were ask to record if they had had receptive anal sex and if so, if they had practiced pre-sex douching, used any rectal sex toys and the type of lubricant (water or silicone based) used. Azithromycin side-effects (nausea, vomiting or diarrhoea) were also collected.

### Specimen collection

A rectal swab was taken at recruitment, prior to the administration of azithromycin. Participants were then asked to collect rectal swabs at 2 hours and 24 hours post dose and then daily on days 2, 3, 4, 7, 10 and 14 (total nine swabs). Swabs were self-collected by inserting the swab (FLOQSwab™, Copan, Italy) 5cm into the rectum and rotating for 5 seconds. A 2mL sample of blood was collected 2 hour post dose as plasma azithromycin levels reportedly peak at 2–3 hours post dose. [15] The blood specimens were centrifuged for 10 minutes at 13000rpm (15000 g) to extract plasma. Swabs were immediately swirled in 1mL 100% methanol (MeOH) with internal standard (IS) (1mcg/mL Leucine enkephalin; Sigma Aldrich Australia) for 20 seconds and stored in a domestic freezer until they were delivered to the laboratory where they were stored at -80°C until analysis. Plasma and tissue samples were analysed using liquid-chromatography tandem mass spectrometry (liquid chromatography-Mass spectrometry; LCMS–see below).

### Specimen preparation

Samples were dried at 30°C and the weight of the tissue was determined by calculating the difference in the weight of the dry Eppendorf tube before and after sample collection. For extraction, 1mL of 100% methanol with IS was added to each dried sample or 50μl of plasma and vortexed for 30 seconds. 1mL of chloroform was then added and vortexed for 1 minute. Samples were then agitated for 30 mins at 20°C and centrifuged at 13000 rpm (15000 g) for 15 mins at room temperature. The supernatant was separated and dried at 30.C. 100μl of 100% methanol was then added and samples were processed on the Agilent QQQ as described below. Lipid concentration was also determined to investigate normalisation of differences in swab collection both within and between participants.

### Liquid chromatography-Mass spectrometry (LCMS)

#### Instrument and conditions

The methods are described in detail in Vodstrcil et al (submitted Plos ONE). In summary the QQQ Mass Spectrometer (MS) (Agilent 6460 LC-MS/Agilent 6490 LC-MS) was used by multiple reaction monitoring (MRM) in positive mode. Chromatography was performed with and Agilent 1290 UPLC system operating at a flow rate of 0.4 mL/min, a column oven temperature maintained at room temperature, an auto sampler maintained at 10°C, and 10μL sample injection. An Agilent Porshell 120 SB-C18 (2.7μm) 2.1 x 100mm column was used, using a binary solvent gradient consisting of water with 5 mM ammonium acetate (buffer A) and acetonitrile (buffer B). The gradient ran from 5% to 80% B from 2 minutes to 4 minutes, then washed the column for 2 minutes with 80% B and re-equilibrated prior to the next injection. MS detection was carried out by MRM transition of *m/z* 749.0 → 591.6 and 556 → 397 for azithromycin and IS respectively.

Extracted lipids were separated by injecting 5μL aliquots of the prepared sample onto a 50mm × 2.1mm × 2.7μm Ascentis Express RP-Amide column (Supelco) using an Agilent LC 1200. Samples were eluted at 0.2mLmin-1 over a 5 min gradient of water/ MeOH/tetrahydrofuran (50:20:30, v/v/v) to water/MeOH/tetrahydrofuran (5:20:75, v/v/v), with the final buffer held for 3 mins. Lipids were analysed by electrospray ionisation-mass spectrometry (ESI-MS) using an Agilent Triple Quad 6460. MS detection was carried out by MRM transition of m/z 760 → 184 to quantify the lipid species of PC(34:1). The capillary voltage, fragmentor voltage, and collision energy were 4000 V, 140–380 V, and 15–60 V, respectively. In all cases, the collision gas was nitrogen at 7 Lmin-1. For all samples and standards, LC-MS data were processed using the Agilent MassHunter quantitative software (version 5).

### Pharmacokinetic analysis

The methods are described in detail in Vodstrcil et al (submitted PloS ONE). In summary for all samples and standards, LCMS data were processed using the Agilent MassHunter quantitative software (Agilent Technology, version 5). The linearity of each calibration curve was determined by plotting the nominal concentration of azithromycin to the peak area ratio of azithromycin, normalised to the IS.

Azithromycin concentrations were then expressed relative to dry tissue weight as mcg/g and examined graphically over time for each participant.

Relative concentrations (mcg/g or mcg/mL) by time, dose, drug side effects, lubricant use, concurrent drug use and tissue type were calculated. In order to assess whether concentrations were adequate to eliminate chlamydia, we assessed effective anti-chlamydial tissue concentrations by measuring the length of time rectal concentrations were above the previously estimated minimum inhibitory concentration (MIC_90_) for chlamydia species of 0.125mcg/mL [23]—assuming mcg/mL being equal to mcg/g in rectal tissue given a conversion ratio of 1.04 has been previously used. [24]

Plasma to tissue ratio after 2 hours post dosing was determined to describe the degree and rapidity of tissue penetration.

A non-compartment pharmacokinetic analysis was performed using Stata (version 13; StataCorp, USA). Total drug exposure was measured as the area under the concentration-time curve (AUC) from time zero to 96 hours (AUC_0-96_) and zero to infinity (AUC_0-∞_) using cubic splines method and expressed as linear of log concentration. Elimination rate constant (Ke) and half-life (t_1/2_), maximum concentrations (Cmax) and time to Cmax (Tmax) were also estimated for each pariticipant

## Results

Results were published in accordance with the Transparent Reporting of Evaluations with Non-randomized Designs (TREND) statement [25] and according to the Reporting Guidelines for Clinical Pharmacokinetic Studies: The ClinPK statement. [26]

### Patient demographics

Ten men were recruited and all provided nine swabs. However two men provided swabs outside of the scheduled times (participant number 8: swab provided on day 13 instead of day 14; participant number 10: swabbed on days 8, 11 and 15 instead of days 7, 10, 14 resepectively). The median age was 41 years (range: 28–55 years) and their median weight and dose by weight (mg/kg) was 75kg (range: 62-111kg) and 13mg/kg (range: 9-16mg/kg) respectively (Table 1).

**Table 1:** Summary of participant demographics, estimated pharmacokinetics parameters, sexual behaviour and concurrent medication use.

Participant	mg/kg	Concentrations throughout study			Time 0–96 hrs		Time 0—Day 14					Comments
		Plasma (mcg/mL) 2 hours post dose	Tissue (mcg/g)	Tissue: Plasma ratio (2 hours)	AUC_0-96_ ((mcg/g).hr)	Elimination half-life (hrs)	AUC_0-∞_ ((mcg/g).hr)	Cmax (mcg/g)	Tmax (hrs)	Ke (per hr)	Elimination half-life (hrs)	
			Range (IQR)^1^									
1	13.9	0.9	11.2–461.4	31.6	20656	16.7	27008	461.4	24	0.0049	140.8	1 episode diarrhoea
			(27.0–131.0)									
2	13.3	1.2	1.2–72.5	1.0	3919	36.4	6321	72.5	24	0.0098	71	1 episode diarrhoea
			(11.0–21.2)									
3	16.1	0.2	0.8–42.4	26.5	1482	21.6	NA	42.4	72	NA	NA	-
			(3.1–20.3)									
4	13.9	1.3	23.9–453.3	342.4	27069	32.9	38463	453.3	2	0.0041	167.2	1 episode diarrhoea
			(24.0–188.0)									
5	12.2	1.4	0.6–35.2	0.5	2509	26.8	3375	35.2	24	0.0093	74.2	-
			(1.4–21.1)									
6	13.5	1.1	3.3–192.6	4.6	NA	NA	9944	192.6	24	0.0081	85.1	
			(7.4–55.4)									
7	13.3	1.2	27.7–2695.8	23.4	NA	NA	28500	2695.8	96	0.0158	43.9	On chronic esomprazole
			(108.0–1524.0)									
8	13.9	0.1	0.3–39.7	5.8	2417	19.5	3474	39.7	48	0.0079	88.1	3 episodes diarrhoea
			(1.9–18.2)									
9	12.5	1.1	1.0–12.7	0.9	3370	354	NA	12.7	24	NA	NA	11 episodes diarrhoea & 4 episodes of using water-based lubricant
			(2.7–6.3)									
10	9.0	1.0	1.1–251.6	1.1	10676	7.8	16262	251.6	48	0.0043	161.5	1 epiode of douching and water-based lubricant use. On chronic tenofovir/ emtricitabine
			(3.6–103.0)									
**Median:**	**13.4**	**1.1**	**-**	**5.2**	**3644.5**	**24.2**	**13103**	**132.6**	**24**	**0.008**	**86.6**	
**Range:**	**9–16.1**	**0.1–1.4**	**0.3–2695.8^1^**	**0.5–342.4**	**1482–27069**	**7.8–354**	**3375–38463**	**12.7–2695.8**	**2–96**	**0.0041–0.0158**	**43.9–167.2**	

(1) excludes pre-dose tissue concentration

AUC = area under the concentration-time curve; Ke = elimination constant; Cmax = maximum concentration; Tmax = Time to Cmax; NA = could not be calculated

Seven men were screened for STIs (including HIV) at the time of recrutiment and all results were negative. Three declined an STI screen because they were in monogomous relationships (n = 2) or had a recent STI screen (n = 1; past week; negative results). Only two participants had receptive anal sex during the study (on or after day 3) with only one performing pre-sex douching.

Six men reported drug side effects–four within 24 hours and one within 48 hours after taking the dose, with most experiencing mild nausea and diarrhoea. One man experienced moderate diarrhoea (11 episodes of diarrhoea in first 72 hours) after taking azithromycin.

Six men were taking regular medicines including one taking long term esomeprazole (gastric acid lowering drug; 20mg once a day) and one taking tenofovir/emtricitabine for HIV pre-exposure prophylaxis (PrEP).

**Fig 2:**
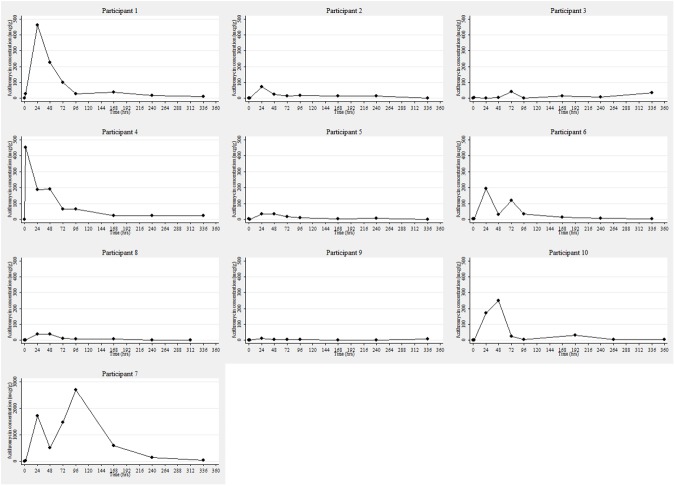
Tissue concentration (mcg/g) over time (hours) by participant*. *The scale of the y-axis for participant 7 is different from all other participants because their azithromycin levels were considerable higher.

Among all men, the median plasma concentration at 2 hours post dose was 1.1mcg/mL (range: 0.1–1.4mcg/mL) and azithromycin tissue concentrations peaked between 2 hours and 4 days (median 24 hours) with a median Cmax of 132.6mcg/g (range: 12.7–2695.8mcg/g) (Table 1). Azithromycin levels remained above the MIC_90_ of 0.125mcg/mL in all men at all times points during the study (Fig 2).

The median tissue to plasma concentration ratio after 2 hours was 5.2 (range: 0.5–342.4). There was no association between median dose per weight of each man and tissue concentrations. For example the 24 hour post dose tissue concentrations was 173, 72.5, 192.6, and 1.9 mcg/g for a 9.0, 13.3, 13.5 and 16.1mg/kg dose (Table 1).

The highest median value over the duration of the study was observed in the man taking esomeprazole, a gut pH-increasing drug (participant 7; median: 1416.1mcg/g; range: 27.7–2695.8mcg/g) and lowest median value was observed in the man who had 11 episodes of diarrhoea over three days followed by 4 episodes of using water-based lubricant during the study (participant 9; median: 3.5mcg/g; range: 1.0–12.7mcg/g). In the man taking esomeprazole, post-baseline tissue concentrations at each time point were considerably higher than median values for all other men (Table 2).

**Table 2:** Azithromycin tissue concentration by esomeprazole use (increased gut pH), douching, moderate diarrhoea and water-based lubricant use.

	Tissue concentration (mcg/g)				
Time	All other men excluding participant 7		Esomeprazole (Participant 7)	Douching and water-based lube^2^	Diarrhoea and water-based lube^5^
				(day 4 only; participant 10)	(day 3,4,10 and 14; participant 9)
	Median	Mean			
**0**	1.3	1.3	2.7	1.3	1.4
				(median: 1.4; mean: 1.3; range:0.2–2.7)	(median: 1.3; mean: 1.3; range: 0.2–2.6)
**2 hours**	3.0	52.5	27.7	1.1	1.0
				(median: 4.9; mean: 58.2; range: 0.3–453.3)	(median: 4.9; mean: 58.2; range: 0.3–453.3)
**Day 1**	122.8	288.3	1707.9	173.0	12.7
				(median: 72.5; mean: 301.1; range:1.9–1707.9)	(median: 173.0; mean: 318.9; range: 1.9–1707.9)
**Day 2**	36.4	132.4	516.6	251.6	5.8
				(median: 33.0; mean: 119.1; range:3.6–516.6)	(median: 39.7; mean: 146.4; range: 3.6–516.6)
**Day 3**	34.1	186.4	1463.1	25.8	5.9^6^
				(median: 42.4; mean: 204.3; range:5.9–1463.1)	(median: 42.4; mean: 206.5; range:11.0–1463.1)
**Day 4**	14.5	286.9	2695.8	3.5^3^	5.3^6^
				(median: 19.5; mean: 318.4; range:0.8–2695.8)	(median: 19.5; mean: 318.2; range: 0.8–2695.8)
**Day 7**	14.4	79.7	598.1	33.0^4^	2.9
					(median: 14.9; mean: 89.4; range: 1.6–598.1)
**Day 10**	8.6	23.8	130.7	3.7^4^	2.4^6^
					(median: 11.6; mean: 26.5; range: 0.3–130.7)
**Day 14**	9.3	15.5	40.8	3.8^4^	7.5^6^
					(median: 11.2; mean: 16.6;range: 0.8–40.8)
**Range**	0.3–461.4^1^		27.5–2670.6	1.1–251.6	1.0–12.7

(1) excludes pre-dose concentration and participant 7.

(2) median, mean and range values for all participants excluding participant 10.

(3) douching and lube use on day 4 only.

(4) swabs taken on days 8,11,15 instead of day 7,10,14. No median, mean, range values for days 8, 11 and 15.

(5) median, mean and range values for all participants except from participant 9; 3–4 episodes of diarrhoea per day for 3 days

(6) Lube used on days 3,4,10 and 14 only

Conversely in the man who experienced moderate diarrhoea and used water-based lubricant, post-baseline tissue concentrations were considerably lower than all the other men.

The estimated AUC_0-96_ and AUC_0-∞_ was 3644 and 13103((mcg/g).hr) respectively. Azithromycin elimination was biphasic in nature with an median initial half life of 24.2 hours (time zero to 96; 0–96) and the total median elimination half life (time zero to day 14) of 86.6 hours. The elimination rate constant was 0.008/hour.

## Discussion

Our LCMS assay found high concentrations of azithromycin in rectal tissue following a single 1g dose, with levels peaking within 24 hours and remaining above the reported MIC for chlamydia for at least 14 days. AUC_0-∞_ was the highest ever reported in human tissue (13103((mcg/g).hr)). This suggests that rectal chlamydia treatment failure is unlikely to be due to poor absorption of aithromycin to the rectal site of infection and the lack of visual correlation between dose and bodyweight (mg/kg) suggest dose adjustment is not required for lower or higher weight individuals.

Tissue concentrations were rapidly attained after 2 hours, peaked after 24 hours and was sustained above the reported MIC for chlamydia species for at least 14 days. These results were comparable to our recent study that found that vaginal levels of azithromycin were rapidly attained after 5 hours, peaked after 48 hours and were sustained above the MIC for up to 9 days after a 1g dose. (submitted PloS ONE [27]) When compared with other sites, we found that azithromycin concentrations were considerably higher in rectal than those previously reported for uterine tissue, cervical mucus, gastric tissue or gastric mucus (Table 3). This suggests that rectal tissue may have a greater capacity to absorb and sustain azithromycin compared with other sites.

**Table 3:** Relative concentrations (mcg/g) by time, dose and tissue type.

	Rectal tissue (1g) (median)	Uterine tissue (0.5g)[28] (mean)	Cervical tissue (0.5g) [28]** (mean)	Cervical mucus (1g)[11] (median)	Gastric tissue (0.5g)[13]* (median)	Gastric mucus (0.5g)[13]* (median)	Gastric tissue (1.5g)[14] (median)
**Day 1**	122.8	1.44	2.8	2.67	3.97	0.48	
**Day 4**	19.5	0.78			4.61	0.47	3.9
**Day 7**	14.4			1.26			
**Day 14**	9.3			0.15			

*day 1 = 24–48 hr post dose and day 4 = 73–96 hr post dose

**day 1 = 17 hr post dose

Our pharmacokinetic data are consistent with our finding of sustained high concentrations of azithromycin in the rectal samples over the duration of follow up. The estimated AUC_0-96_ and AUC_0-∞_ were 3644 and 13103((mcg/g).hr) respectively. This AUC_0-∞_ is a very high result and is considerably higher than that previously reported for polymorphonuclear leukocytes (6067((mcg/mL).hr)) following a 1g dose [29] and that in lung tissue following a 3g dose (2514((mcg/g).hr)). [30] No comparative data are available however, for urogenital tissue.

Azithromycin elimination was biphasic in nature with an median initial half life of 24.2 hours (time zero to 96; 0–96). The total median elimination half life (time zero to infinity; 0-∞) was 86.6 hours which is higher than the 60, 67, 68 and 77 hours reported for prostatic, [31] gynaecological, [12] serum, [15] and tonsilar [10] tissue respectively. The elimination rate constant was 0.008/hr, lower than reported values for a 500mg dose for prostatic (0.0104/hr) [31] and gynaecological tissue (0.0116/hr). [12] The slower rate of elimination and longer half life we observed in this study explains the sustained high levels over the duration of follow up in this study.

Given that we observed high levels of azithromycin over time, this suggests that other factors could affect rectal concentration *in situ*. Firstly low tissue concentrations could result from poor drug absorption (e.g. from diarrhoea) or factors that deplete azithromycin from tissue due to loss of epithelial tissue such as pre-sex douching [19] and water-based lubricants. [20] This was seen in participant 9 who reported 11 episodes of diarrhoea within 72 hours of taking azithromycin, immediately followed by four episodes of water-based lubricants use during receptive anal sex between days 3–14. This man reported the lowest concentrations (median: 3.5mcg/g; range: 1.0–12.7mcg/g) compared to the other men. This suggests drug absorption from the gut may directly correlate with tissue concentrations but these data are not routinely reported with only two studies [32, 33] in a recent meta-analysis of rectal chlamydia treatments [5] excluding cases who reported diarrhoea in their azithromycin efficacy estimates. While diarrhoea could reduce tissue concentrations through malabsorption it is possible that multiple episodes of diarrhoea may also ‘wash’ away drug containing rectal tissue and might have been compounded by, and have similar negative effects, to the use of water-based lubricants or douching. Similarly the excess fluids in the rectum may have resulted in a dilutional effect on the measured concentrations. One man in our study reported pre-sex douching in our study and he recorded lower concentrations at day 4 compared to the man reporting diarrhoea/water-based lubricant (day 4 concentration: 3.5 vs 5.3 mcg/g respectively). However it may be possible that receptive anal sex itself may have resulted in loss of rectal tissue due to physical trauma, independent of lubricant use, thereby, depleting rectal levels of azithromycin.

Another factor that may contribute to azithromycin’s concentrations and efficacy is the environmental pH in which azithromycin exists. In our study, the man with the highest tissue concentrations was on long term esomeprazole (median: 1416.1mcg/g; range: 27.7–2695.8mcg/g). Esomeprazole raises the intra-gastric pH by reducing acid production [34] and may possibly have an effect on rectal pH or on the absorption of azithromycin from the gut resulting in higher intracellular concentrations. Azithromycin has a pKa of ~8.5 [15] meaning at a pH of 8.5, 50% of the drug is ionised and 50% is unionised. The unionised form is important because this form can permeate across cellular membranes and enter a cell, [22] contributing to intracellular concentrations. A one unit increase in the pH from the pka results in 91% of the drug being unionised, while a one unit decrease results in only 9% being unionised [35]–the former potentially improving drug efficacy. The optimal effects of macrolides have been suggested to be at a pH of 8 with a significant decrease in its efficacy at pH values <6. [36] Therefore higher pH levels may be associated with greater efficacy and tissue penetration than lower pH levels. The pH of the human rectum has been reported to decrease due to inflammatory disease [37] but nothing is known whether inflammation from chlamydia infection could result in a similar drop in pH thereby reducing treatment efficacy.

There are several limitations to our study. Firstly, the small sample size reduces the generalisability of our results to men more broadly and the precision of our estimates. Secondly, faecal, blood and rectal mucus contamination could have affected our results. Blood levels of azithromycin have been shown to be undetectable after 24 hours [13] or present at very low levels [38] so blood contamination is more likely to affect concentrations measured within 24 hours post dose, such as what might have been seen in participant 4 (2 hour post dose tissue concentration of 453.3mcg/mL vs median of 3.0mcg/mL for other participants). Approximately 47% of azithromycin is excreted in faeces in the first 24 hours [18] so this could affect samples taken within 48 hours of taking azithromycin but is less likely to impact on results after this time. However, azithromycin in faeces may have a beneficial antimicrobial effect as it could have a localised drug effect since it would have direct contact with rectal mucosal/epithelial tissue. Similarly contamination of azithromycin in rectal mucus may have also contributed to our results. While no data exist on azithromycin levels in rectal mucus, azithromycin has been reported in cervical mucus [11] and gastric mucus [13] so it remains plausible that drug would be present in rectal mucus. Thirdly, high tissue concentrations may not always translate to clinical efficacy due to the relative distribution of azithromycin between different tissue compartments, [39] particularly under the influences of pH [40, 41] and that intracellular concentrations may be potentially unavailable for activity. [41] However despite these pharmacokinetic limitations, observational studies have reported an efficacy of 83% for 1g azithromycin for treating rectal infections [5], supporting the likelihood that reasonable rectal tissue concentrations are being obtained following a 1g dose with the results of this study representing the only published study to date that has quantified rectal concentrations against treatment efficacy. Also only protein-unbound (“free”) drug is pharmacologically active. [42] Protein binding for azithromycin is low and concentration dependent, decreasing from 51% at 0.02mcg/mL to 7% at 2mcg/mL, [15] which suggests at high concentrations, protein binding may be saturated resulting in more free drug. At the reported concentrations in our study (86% samples being >2 mcg/g), most of drug would be expected to be “free” drug. Ideally rectal biopsy and measurement of “free” drug as previously described [43] would have yielded more accurate results but this would have been costly and medically invasive. Fourthly, we used self-collected rectal swabs as a surrogate for rectal tissue concentration. However, we measured the dry tissue weight of the specimen and the level of azithromycin was standardised by calculating its concentration relevant to tissue weight. We also used internal standards to account for any sampling variability between participants. Fifthly, the two participants who declined to have an STI screen may have had an STI which may have affected the results. However it is likely that if they had an infection, the inflammation that ensued would most likely have increased tissue concentrations compared to non-inflammed tissue. [44] Lastly samples were stored in a domestic freezer prior to analysis and degradation may have occurred. However, azithromycin appears to be extremely stable at high and low concentrations under a range of testing conditions [45, 46] for up to 25 days. [47] Nevertheless, this study provides the only available evidence of azithromycin pharmacokinetics in rectal tissue and allows some initial discussions on possible factors that could affect concentrations *in situ*.

## Conclusion

Rectal concentrations of azithromycin following a single 1g dose are high and above the MIC for chlamydia species for at least 14 days in most situations and unlikely to contribute to treatment failure. Other factors that could effect concentrations *in situ* (douching, malabsorption or rectal pH) may be responsible and collecting this data in the context robust RCTs are needed to clarify the extent to which these factors contribute to treatment failure. If rectal chlamydia infections are shown to reduce rectal pH and azithromycin efficacy, trials of extended doses of azithromycin should be tested to see if this can overcome treatment failures.

## Supporting information

S1 Table TREND statement checklist. (PDF)

S2 Table CLINPK checklist. (PDF)

S1 Text Study Protocol. (DOCX)
