# Supplementary material for: Pharmacokinetics of a single 1g dose of azithromycin in rectal tissue in men
Source: PLoS One. 2017 Mar 28;12(3):e0174372. doi: 10.1371/journal.pone.0174372 (PMC5370104; doi:10.1371/journal.pone.0174372)
Supplement: S2 Table — (PDF) [file pone.0174372.s002.pdf]

Section	Checklist Item Number	page number	Checklist Item
Title/abstract	1	1	The title identifies the drug(s) and patient population(s) studied.
	2	2	The abstract minimally includes the name of the drug(s) studied, the route of administration, the population in whom it was studied, and the results of the primary objective and major clinical pharmacokinetic findings.
Background	3	4	Pharmacokinetic data (i.e., absorption, distribution, metabolism, excretion) that [are] known and relevant to the drugs being studied [are] described.
	4	4	An explanation of the study rationale is provided.
	5	4	Specific objectives or hypotheses [are] provided.
Methods	6	5	Eligibility criteria of study participants are described.
	7	5,10,11	Co-administration (or lack thereof) of study drug(s) with other potentially interacting drugs or food within this study is described.
	8	5	Drug preparation and administration characteristics including dose, route, formulation, infusion duration (if applicable), and frequency are described.
	9	6	Body fluid or tissue sampling (timing, frequency, and storage) for quantitative drug measurement is described.
	10	7	Validation of quantitative bioanalytical methods used in the study [is] referenced or described if applicable.
	11	8,9	Pharmacokinetic modeling methods and software used are described, including assumptions made regarding the number of compartments and order of kinetics (zero, first, or mixed order).
	12	NA	For population pharmacokinetic studies, covariates incorporated into pharmacokinetic models are identified and described.
	13	NA	Formulas for calculated variables (such as creatinine clearance, body surface area, AUC, and adjusted body weight) are provided or referenced.
	14	10	The specific body weight used in drug dosing and pharmacokinetic calculations [is] reported (i.e., ideal body weight versus actual body weight versus adjusted body weight).
	15	8,9	Statistical methods including software used are described.
Results	16	NA	Study withdrawals or subjects lost to follow-up (or lack thereof) are reported.
	17	9	Quantification of missing or excluded data is provided if applicable.
	18	9,10,11	All relevant variables that may explain inter- and intra-patient pharmacokinetic variability (including: age, sex, end-organ function, ethnicity, weight or BMI, health status or severity of illness, and pertinent co-morbidities) are provided with appropriate measures of variance.
	19	NA	Results of pharmacokinetic analyses are reported with appropriate measures of precision (such as range or 95% confidence intervals).
	20	NA	Studies in patients receiving extracorporeal drug removal (i.e., dialysis) should report the mode of drug removal, type of filters used, duration of therapy, and relevant flow rates.
	21	NA	In studies of drug bioavailability comparing two formulations of the same drug, F (bioavailability), AUC, Cmax (maximal concentration), and Tmax (time to maximal concentration) should be reported.
Discussion/conclusion	22	16,17,18	Study limitations describing potential sources of bias and imprecision where relevant should be described.
	23	13-18	The relevance of study findings (applicability, external validity) is described.
Other information	24	19	Funding sources and conflicts of interest for the authors are disclosed.
