# Supplementary material for: Pharmacokinetics of a single 1g dose of azithromycin in rectal tissue in men
Source: PLoS One. 2017 Mar 28;12(3):e0174372. doi: 10.1371/journal.pone.0174372 (PMC5370104; doi:10.1371/journal.pone.0174372)
Supplement: S1 Text — (DOCX) [file pone.0174372.s003.docx]

**Pharmacokinetics of azithromycin 1 gram single dose in anorectal tissue and serum**

**Version 1.2**

**12/6/2015**

**Submitted to ethics: 22/5/2015**


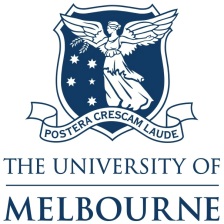

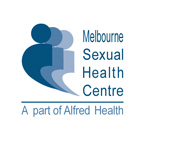

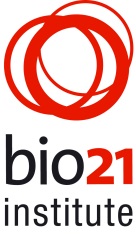

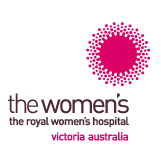


**PROTOCOL COVER SHEET**

| Study title | Pharmacokinetics of azithromycin 1g single dose in anorectal tissue and serum | |
| --- | --- | --- |
| Version | Version 1.0  27^th^ April 2015 | |
| Study sites | Melbourne Sexual Health Centre (MSHC) | |
| Study Initiation date | 2015 | |
| Collaborating institutions | The University of Melbourne (UoM)  Melbourne Sexual Health Centre (MSHC)  Bio21, The University of Melbourne (Bio21, UoM)  Royal Women’s Hospital (RWH) | |
| Sponsor | University of Melbourne | |
| Principal investigator (PI): | A/Prof Jane Hocking | |
| Associate Investigators: | A/Prof Julie Simpson  Mr Fabian Kong  Professor Christopher Fairley  A/Prof Marcus Chen  A/Prof Catriona Bradshaw  Dr Lenka Vodstrcil  Dr Thusitha Rupasinghe  Dr Dedreia Tull  Professor Malcolm McConville  A/Prof Sepehr Tabrizi | UoM  UoM  MSHC, Monash University  MSHC  MSHC  UoM, MSHC  Bio21, UoM  Bio21, UoM  Bio21, UoM  RWH |

Table of Contents

[INVESTIGATORS: 4](#_Toc416696165)

[BACKGROUND 7](#_Toc416696166)

[PROTOCOL 9](#_Toc416696167)

[Overall aim 9](#_Toc416696168)

[Duration of study 9](#_Toc416696169)

[Methods 9](#_Toc416696170)

[Inclusion criteria 9](#_Toc416696171)

[Exclusion criteria 9](#_Toc416696172)

[Specimen sampling 9](#_Toc416696173)

[Follow up 10](#_Toc416696174)

[Data collection 10](#_Toc416696176)

[Pharmacokinetic and statistical analysis 11](#_Toc416696185)

[ETHICS 12](#_Toc416696192)

[PROPOSED BUDGET 13](#_Toc416696195)

[APPENDIX 1 - Pharmacokinetic considerations 14](#_Toc416696196)

[APPENDIX 2 - Pharmacokinetic of azithromycin in vaginal tissue 24](#_Toc416696197)

[REFERENCES 27](#_Toc416696198)

# INVESTIGATORS:

| A/Prof Jane Hocking  Centre for Epidemiology and Biostatistics  Melbourne School of Population Global Health  University of Melbourne  Level 3, 207 Bouverie St  Carlton 3053, Victoria  Tel: +61 3 8344 0762  [j.hocking@unimelb.edu.au](mailto:j.hocking@unimelb.edu.au) | A/Prof Julie Simpson  Centre for Epidemiology and Biostatistics  Melbourne School of Population Global Health  University of Melbourne  Level 3, 207 Bouverie St  Carlton 3053, Victoria  Tel: +61 3 8344 0732  [julieas@unimelb.edu.au](mailto:julieas@unimelb.edu.au) |
| --- | --- |
| Mr Fabian Kong  Centre for Epidemiology and Biostatistics  Melbourne School of Population Global Health  University of Melbourne  Level 3, 207 Bouverie St, Carlton 3053, Victoria  Tel: +61 3 9035 3039  [kongf@unimelb.edu.au](mailto:kongf@unimelb.edu.au) | Professor Christopher Fairley  Melbourne Sexual Health Centre  Monash University  580 Swanston St  Carlton 3053, Victoria  Tel: +61 3 9341 6231  [cfairley@mshc.org.au](mailto:cfairley@mshc.org.au) |
| A/Prof Catriona Bradshaw  Melbourne Sexual Health Centre  580 Swanston St  Carlton 3053, Victoria  Tel: +61 3 9341 6253  [CBradshaw@mshc.org.au](mailto:CBradshaw@mshc.org.au) | Dr Lenka Vodstrcil  Melbourne School of Population Global Health  University of Melbourne  580 Swanston St  Carlton 3053, Victoria  Tel: +61 3 9341 6232  [lv@unimelb.edu.au](mailto:lv@unimelb.edu.au) |
| Associate Professor Marcus Chen  Melbourne School of Population Global Health  University of Melbourne  580 Swanston St  Carlton 3053, Victoria  Tel: +61 3 9341 6260  [mchen@mshc.org.au](mailto:mchen@mshc.org.au) | Professor Malcolm McConville  Deputy Director  Bio21 Institute  Building 102, 30 Flemington Road Parkville, Victoria, Australia, 3010 Tel: [+61 3 8344 2342](tel:%2B613%208344%202487)  [malcolmm@unimelb.edu.au](mailto:malcolmm@unimelb.edu.au) |
| Dr Thusitha Rupasinghe Metabolomics Australia Bio21 Institute  Building 102, 30 Flemington Road Parkville, Victoria, Australia, 3010 Tel: [+61 3 8344 2487](tel:%2B613%208344%202487) [tru@unimelb.edu.au](mailto:tru@unimelb.edu.au) | Dr Dedreia Tull Metabolomics Australia Bio21 Institute  Building 102, 30 Flemington Road Parkville, Victoria, Australia, 3010 Tel: [+61 3 8344 2220](tel:%2B613%208344%202487) [dedreia@unimelb.edu.au](mailto:dedreia@unimelb.edu.au) |
| A/Prof Sepehr Tabrizi  Senior Research Scientist  Department of Microbiology  Royal Women’s Hospital  University of Melbourne, Carlton 3053, Vic  Tel: +61 3 8345 3672  [Sepehr.Tabrizi@thewomens.org.au](mailto:Sepehr.Tabrizi@thewomens.org.au) |  |

**INVESTIGATORS’ EXPERTISE**

**Associate Professor Jane Hocking**

Associate Professor Hocking is one of Australia’s leading chlamydia epidemiologists and she heads up the Sexual Health Unit within the School of Population and Global Health at University of Melbourne. She generated Australia’s first population estimates of chlamydia prevalence, incidence and re-infection rates among young women and is currently leading a cohort study of 300 women infected with chlamydia that aims to determine whether chlamydia treatment failure after treatment with 1 gram azithromycin is an issue.

**Associate Professor Julie Simpson**

Julie Simpson is an Associate Professor of biostatistics at the University of Melbourne. Her main area of expertise is the design and analysis of population pharmacokinetic studies, particularly for drugs used to treat malaria. Julie has participated in international committees advising on the planning of worldwide malaria studies and has been an advisor to World Health Organisation for the design of new antimalarial drug trials. She is currently a member of the Data Safety Monitoring Committee of three large international studies and a chartered statistician of the Royal Statistical Society, UK.

**Mr Fabian Kong**

Mr Kong is a hospital trained pharmacist with over 15 years of public health experience in the combined fields of clinical pharmacy, harm reduction and toxicology. He has published widely in the area of clinical pharmaceutics. Fabian also holds a Masters of Epidemiology with over 8 years of research experience and has worked as a pharmaceutical specialist in International Health since 2002 predominantly with UN agencies (WHO, UNDP) and The World Bank. He is currently undertaking his PhD examining rectal chlamydia treatment failure.

**Professor Christopher Fairley**

Professor Fairley is Australia’s leading sexual health physician and Director of the Melbourne Sexual Health Centre (MSHC). He holds three specialist medical fellowships from the College of Physicians, Faculty of Public Health and the Chapter of Sexual Health Medicine. He is also a member of the International Executive of the International Union of Sexually Transmitted Infections. He chaired the 2006 and 2012 Sexual Health Conference organising committee and was involved in the organisation of the 2007 International Sexually Transmitted Diseases Committee in Seattle. He is an editor of the Journal ‘Sexual Health’ and his principal research interest is the public health control of sexually transmitted infections.

**Dr Lenka Vodstrcil**

Dr Vodstrcil is a postdoctoral researcher with molecular microbiology and public health research experience. Since completing her PhD researching the roles of relaxin and its receptors in the female reproductive tract during pregnancy, her research has focused on a variety of sexual and reproductive health projects including the epidemiology of chlamydia and bacterial vaginosis. In consultation with Dr Thusitha Rupasinghe, Lenka developed an assay for detecting azithromycin in self collected vaginal swabs.

**Associate Professor Marcus Chen**

Associate Professor Chen is the clinical director at MSHC with a PhD in epidemiology. He is Fellow of the Australasian Chapter of Sexual Health Medicine, Royal Australasian College of Physicians. He is a sexual health physician with a strong understanding of the epidemiology of chlamydia infection. He has particular research interest in treatment efficacy and antimicrobial resistance and is the lead investigator on a randomised clinical trial comparing different treatment regimens for gonorrhoea infection.

**Associate Professor Catriona Bradshaw**

A/Prof Bradshaw is a Catriona is a senior specialist in sexual health medicine at Melbourne Sexual Health Centre and The Alfred Hospital. She is a Fellow of the Australasian Chapter of Sexual Health Medicine and a member of the Royal Australian College of Physicians after completing specialist training in Sexual Health (1998-2002). A/Prof Bradshaw in an international expert in the epidemiology of bacterial vaginosis and non-gonococcal urethritis.

**Dr Thusitha Rupasinghe, Dr Dedreia Tull and Professor Malcolm McConville of Bio21**

Metabolomics Australia of Bio21 was established in 2007 and is a research service-delivery consortium that offers high throughput metabolomics services to life sciences researchers in academia and industry. It was established with funding from the Federal Government National Collaborative Research Infrastructure Strategy (NCRIS) scheme to Bioplatforms Australia Pty Ltd and co-investment from State governments and institution partners. The facility offers access to expertise and technologies that cover a wide range of metabolite chemistries and quantitative analyses required for comprehensive metabolite profiling applicable to biomedical, agri-food and environmental sciences.

Dr Rupasinghe is a senior research scientist at Metabolomics Australia with expertise in liquid chromatography mass spectrometry (LCMS) platform for small molecule and lipid analysis using targeted and untargeted approach. Dr Rupasinghe has undertaken a similar pilot study of azithromycin pharmacokinetic in cervical tissue as part of the Australian Chlamydia Treatment Study (ACTS) (<http://www.biomedcentral.com/1471-2334/13/379>).

Dr Dedreia Tull is a biochemist who specializes in the research and management of analytical projects ranging from biotechnological and agricultural to biomedical. She works across academic, government to commercial institutions. Dedreia leads the analytical team and contributes to the management of the bioinformatics team at Bio21 Institute Metabolomics Australia.

Malcolm McConville is the Professor of Biochemistry and Molecular Biology at the University of Melbourne and Head of Bio21 Institute node of Metabolomics Australia. He has a long-standing interest in the metabolism of microbial pathogens with the view of identifying new drug targets. His work at Bio21 is directed at understanding how microbial pathogens survive within their mammalian hosts with the view of identifying new therapies including ne antimicrobial agents and vaccines.

**Associate Professor Sepehr Tabrizi**

Associate Professor Tabrizi is the chief scientist within the Department of Molecular Microbiology at the Royal Women’s Hospital. His research has had a strong focus on the using molecular tools for better understanding disease transmission and epidemiology of STIs as well as clinical translation of research findings. This has led to improvements in the diagnosis, management and prevention of STIs. He has developed novel patient collected non-invasive sampling techniques for detecting STI pathogens; this has led to greatly improved STI detection and has been widely used in STI research internationally.

# BACKGROUND

*Chlamydia trachomatis* is the most common bacterial sexually transmitted infection (STI) worldwide. [[1](#_ENREF_1)] In countries that target both men and women for screening, approximately 40% of chlamydia diagnoses are among men [[2-5](#_ENREF_2)] and while these data do not differentiate between rectal, urethral or other sites of infection, available prevalence data suggest that among men who have sex with men (MSM), the prevalence of rectal chlamydia is higher than urethral infection, with prevalence estimates for rectal chlamydia ranging from 3% to 13% in MSM. [[6-10](#_ENREF_6)]

Rectal STIs are an important public health issue because of the increased risk of HIV acquisition and transmission [[11-13](#_ENREF_11)] with high HIV RNA being found in the rectal mucosa [[14](#_ENREF_14)] and repeat rectal chlamydial infections being associated with increased risk of HIV infections. [[15-17](#_ENREF_15)] Repeat rectal STI infections are not uncommon with approximately 14% of Australian MSM who received post-exposure prophylaxis (PEP) for the prevention of HIV re-engaging in unprotected rectal intercourse within two weeks of receiving treatment. [[18](#_ENREF_18)] For this reason, highly efficacious treatment is needed to break the ongoing transmission of infection.

Current testing guidelines for MSM in the United States and Australia recommend testing rectal swabs for chlamydia using nucleic acid amplification tests (NAAT) and treatment of infection diagnosed with a single 1g dose of azithromycin or 7 days of doxycycline (100mg twice daily) [[19](#_ENREF_19)] with doxycycline being less preferred because of compliance issues. [[20](#_ENREF_20)] However, there are increasing concerns about the effectiveness of azithromycin for treating rectal chlamydia [[21](#_ENREF_21)] with studies reporting treatment failure rates ranging from 13% to 21%. [[22-25](#_ENREF_22)] In response to these concerns, the current European guidelines recommend positive rectal infections be treated with 7 days of doxycycline.

A recent meta-analysis of randomised controlled trials (RCTs) comparing azithromycin 1g single dose with doxycycline 100mg twice daily for 7 days for the treatment of *urogenital* chlamydia infections showed up to a 3% efficacy difference between these treatments in favour of doxycycline (azithromycin and doxycycline treatment efficacy of 94.3% and 97.1% respectively). [[26](#_ENREF_26)] However, there were limitations in the available evidence (only 4 of the 23 trials included in the meta-analysis were double-blinded) suggesting that there may not be any real difference in efficacy between the two treatments. A similar meta-analysis was undertaken to investigate treatment efficacy for *rectal* chlamydia infection. This meta-analysis found that doxycycline had greater efficacy than azithromycin (99.6% and 82.9% respectively), [[27](#_ENREF_27)] but the quality of studies included was very poor with no RCTs comparing the two treatments.

The available pharmacokinetic data for azithromycin in cervical tissue suggest that azithromycin should remain at high enough levels to kill chlamydia for up for to 14 days following a single 1 gram dose [[28](#_ENREF_28), [29](#_ENREF_29)]. However, there are no pharmacokinetic data available for azithromycin in rectal tissue and given that the microbiome and immune response in the rectal mucosa may be weaker [[30](#_ENREF_30)] than that observed in the cervix and vagina, drugs such as anti-retrovirals have been shown to have markedly different affinities for vaginal and rectal tissue [[31-33](#_ENREF_31)] and that there are increasing concerns about treatment efficacy for rectal chlamydia, it is possible that azithromycin may have different antimicrobial actions in the rectum. Further, it may be that different dosing regimens are required for treating rectal chlamydia infections. (See Appendix 1 for pharmacokinetic considerations)

In response to concern about increasing levels of antimicrobial resistance, the WHO has recently classified macrolides as critically important antimicrobials requiring ongoing susceptibility surveillance and judicious use to maintain their effectiveness in humans. [[34](#_ENREF_34)] Given the concern about potential azithromycin treatment failure for rectal chlamydia infection, increasing HIV rates and high rectal chlamydia prevalence in MSM, pharmacokinetic data for azithromycin in rectal mucosa are urgently needed. This protocol describes the methodology for a pharmacokinetic study of azithromycin in rectal tissue among men who have sex with men.

# PROTOCOL

## Overall aim

To determine the key pharmacokinetic properties of a single 1g oral dose of azithromycin in rectal tissue.

## Duration of study

Recruitment will commence in July 2015 and expected to continue until December 2015 with results reported in mid-2016. Participants will be followed up for 14 days after taking a single 1 gram dose of azithromycin.

## Methods

Up to 20 men who have sex with men (MSM) will be recruited. Recruitment will be by advertising through MSHC, at University of Melbourne or social media. Eligible men will then be referred to the research nurse at MSHC who will again explain the project and obtain informed consent via the PICF in a private office at MSHC and screen the man for eligibility. Researchers referring men for recruitment will not have a personal relationship with the person being referred.

Once informed consent is obtained the men will be screened for eligibility. Participants will then be asked to complete a survey and a rectal swab will be collected. They will be then given a directly observed 1 gram oral dose of azithromycin. Participants will then be followed up with further specimen collection as described below (see Table 1).

## Inclusion criteria

- Men who are HIV/STI free and chlamydia negative
- Men aged 18 years and over
- Adequate English and comprehension skills to give informed consent

## Exclusion criteria

- Self-report of antibiotic use in the last 2 weeks
- Current commercial sex work
- Men who do not have a mobile phone
- HIV positive status
- Concurrent medication likely to significantly interact with azithromycin (e.g. cyclosporine, digoxin)
- Known macrolide allergy or contraindications

## Specimen sampling

Self-collected rectal swabs will be collected and used as a surrogate for rectal tissue. Based on previous studies following a single 1 g dose, peak blood levels were reported 2 hours post dose. [[35](#_ENREF_35), [36](#_ENREF_36)] As no previous studies in rectal tissue has been reported, azithromycin concentrations in gastric tissue was used as a surrogate for rectal tissues with studies reporting peak gastric tissue concentrations between 3-5 days post dose. [[37](#_ENREF_37), [38](#_ENREF_38)]

The research nurse will provide education to participants on how to self-collect rectal swabs in a standardised manner. Swabs will be taken from the rectum no further than 10cm from the rectal verge as has been done in other pharmacokinetic studies in rectal mucosa. [[39-42](#_ENREF_39)] A total of one blood sample and 9 rectal swabs will be collected over 14 days for each man. See Table 1 for specimen sampling strategy. The research nurse will collect the blood specimen at 2 hours after taking azithromycin. For specimen collection after day 1, participants will be asked to self-collect each rectal swab at the same time each day.

Both rectal swabs and blood samples will be used to measure azithromycin concentrations.

Table 1: Sampling strategy

|  | Hours after treatment | | | Days after treatment | | | | | |
| --- | --- | --- | --- | --- | --- | --- | --- | --- | --- |
| Clock  time | 0900 | 1100 | 0900 | 0900 | 0900 | 0900 | 0900 | 0900 | 0900 |
| Time  (post dose) | 0^1^ | 2.0 | 24 | 2 | 3 | 4 | 7 | 10 | 14 |
| Swab | X | X | X | X | X | X | X | X | X |
| Blood^2^ |  | X |  |  |  |  |  |  |  |

^1^ Collected prior to taking treatment^2^; Blood (4mL) with corresponding swab at 2 hours;

All swabs for azithromycin testing will be immediately placed in cold 100% methanol and placed at -80.C freezer MSHC or in a domestic freezer if collection is at participants home. All samples will then be transferred to Bio21 for analysis.

## Follow up

Participants will be followed up over the 14 day study period by a call or SMS and/or email from the research nurse.

Participants will be reimbursed for their time and/or transport cost: $50 for baseline visit at MSHC and $50 after providing the last rectal swab sample. Total payment will be $100 provided as Coles/Myer vouchers. We have previously shown that this level of payment is necessary to ensure participants remain in the study and provide the required swabs. It is vital that we minimise any loss to follow up. This study does place a considerable burden on each participant and reimbursement is necessary to help minimise the impact of this burden.

## Data collection

Participants will be asked to provide the following data via a survey administered at recruitment:

- age,
- height,
- weight,
- any medications used,
- whether participants practiced rectal douching or had any sex (including the use of intra-rectal devices eg. toys) in the last 24 hours,
- type of lubricant (silicone or water based^[[1]](#footnote-1)^) used if they have had sex.

With each swab, participants will be asked whether they have had any adverse events (vomiting, diarrhoea), practiced rectal douching or had any sex (including the use of intra-rectal devices eg. toys) since the last swab collected and type of lubricant (silicone or water based^[[2]](#footnote-2)^) used if had sex.

## Pharmacokinetic and statistical analysis

*Measurement of tissue concentrations*

Azithromycin levels as well as its metabolites will be measured using LCMS (Liquid Chromatographic Mass Spectrometry) at Metabolomics, Bio21, University of Melbourne. We have previously measured azithromycin levels over time in self collected high vaginal swabs and will be using similar methodology here (see Appendix 2 for further detail). In brief, absorption concentration over time will be monitored for each participant. Protein unbound (free) drug concentrations will be measured where possible as free drug correlates with greater tissue penetration and is the biologically active form of the drug [[43](#_ENREF_43), [44](#_ENREF_44)] (see Appendix 1). Additionally as chlamydia infections and azithromycin are found intracellularly, intracellular drug concentrations will be measured where possible. Lastly, as a large proportion (~47%) [[45](#_ENREF_45)] of the oral dose is excreted unchanged in faeces, methods to minimise faecal contamination of samples will be piloted by excluding samples which are visually contaminated and improving extraction methods. This will be part of the validation process in developing a novel method for measuring azithromycin in rectal samples.

For all samples and standards, LCMS data will be processed using the Agilent Mass Hunter quantitative software (version 5).

*Pharmacokinetic (PK) analysis*

First, the observed concentration versus time data will be presented as a spaghetti plot to visually assess the concentration-time relationship within an individual. Second a standard two-stage classical PK analysis will be performed. In stage 1, compartmental pharmacokinetic models (one and two-compartment PK models with first order absorption) will be fitted to each individual’s drug concentration versus time data to determine the best PK model that describes the concentration versus time relationship. The PK parameters will be estimated using nonlinear regression with the minimization method of ordinary least squares and the Gauss-Newton searching algorithm. The goodness of fit of each PK model to the individual concentration-time data will be assessed visually by superimposing the predicted concentrations on the graph of observed concentrations versus time, by examining the residual plot and statistically by using the Akaike Information Criterion (AIC) and the precision of the parameter estimates. In stage 2, individual estimates of the PK parameters (e.g. for a one compartment model the PK parameters are absorption rate constant, apparent volume of distribution and apparent clearance) will be extracted as well as posthoc calculation of the parameters, Cmax (maximum serum concentrations), Tmax (time at maximum concentration) and area under the concentration-time curve (AUC_0-∞_). Descriptive statistics (mean (SD) for normally distributed data and median (25^th^ – 75^th^ percentiles) for non-normally distributed data) will be reported to describe the distribution of the PK parameter estimates for the 20 study participants.

The area under the concentration curve for the standard curve and each participant at all time-points for azithromycin, the internal standard (IS) and lipid species 34:1, will be estimated. Using the equation generated by the standard curve of each run, the concentration of azithromycin in each sample will be calculated.

A value of zero will be used as the initial mean and median concentration at time zero for determination of the area term of serum and tissues.

It will be assumed that the majority of azithromycin will excreted unchanged in bile with no active metabolites as per the product information. [[46](#_ENREF_46)]

The elimination rate constant of azithromycin will be only determined for patients providing data for at least 85% (12 of 16) of swabs over the sampling period from baseline to day 14.

Correlations with patient 7,10 will be assessed. Comparisons between azithromycin levels and the minimal inhibitory concentration (MIC) for chlamydia will be investigated using available MIC data. [[47](#_ENREF_47)]

Peak serum concentrations (2 hours post dose) will be used to calculate plasma to tissue ratios to determine the extent of drug penetration in rectal tissue. For example the concentration ratio was 70 for cervical tissue measured at 19 hours after a 500mg dose. [[46](#_ENREF_46)]

Pharmacokinetic analysis will be carried out using STATA (version 13.0; StataCorp, College Station, TX, USA).

# ETHICS

Ethics approval will be sought from the Alfred Hospital Human Research Ethics Committee prior to the commencement of the study.

We have previously undertaken a similar study investigating azithromycin levels in self collected high vaginal swabs (Australian Chlamydia Treatment Study (ACTS; #480/11, Alfred Hospital Human research Ethics Committee).

## Dissemination of results

Results of this study will be submitted for publication in a peer reviewed journal and presented at national and international conferences.

# PROPOSED BUDGET

The total financial support being requested is $22,167.20 inclusive of GST. See below for breakdown of costs.

| Item | Description | Amount |
| --- | --- | --- |
| Azithromycin 500mg tablets | Treatment | 20 participants x 1g (2 tablets) = 40 tablets @ $1 per 1g dose. Total $20 |
| Research nurse | For participant recruitment, handling of swabs, data collection and management, follow up. | As part of normal duties |
| Incentive payments | $100 per participant | $2000 |
| Testing of blood and tissue concentrations | $80 per sample x 10 samples (1 blood and 9 swabs) x 20 participants | $16,000 |
| Swabs/collection kits | $15 per participant | $300 |
|  | ***subtotal*** | ***$18,320*** |
|  | *UoM Indirect costs 10%* | *$1,832* |
|  | *Subtotal (ex GST)* | *$20,152* |
|  | *GST* | *$2015.20* |
|  | **TOTAL (incl. GST)** | **$22,167.20** |

# APPENDIX 1 - Pharmacokinetic considerations

Below is a summary of the literature regarding potential confounders that could influence the rectal pharmacokinetics of azithromycin.

The pharmacokinetics of azithromycin are best described by a 3-compartment pharmacokinetic model [[48](#_ENREF_48)] with the drug possessing a low oral bioavailability of 37%, long terminal half-life of 68 hours and high volume of distribution of 31.1 L/kg. [[46](#_ENREF_46)] The serum protein binding of azithromycin is low and concentration dependent, decreasing from 51% at 0.02 microgram/mL to 7% at 2 microgram/mL [[29](#_ENREF_29), [46](#_ENREF_46)] which suggest at high serum concentrations, protein binding is saturated resulting in more free unbound drug. This is likely as azithromycin is predominantly bound to alpha-1 acid glycoprotein (AGP), [[29](#_ENREF_29)] which is found in considerable lower concentrations compared to the other important serum protein, albumin (0.015nM vs 0.6nM). [[49](#_ENREF_49)] However this was not the case with an *in vitro* study showing that azithromycin was only ~30% bound to AGP and that saturation of binding sites occurred at only high drugs concentrations than would probably not be expected to be achieved from a standard oral dose. [[50](#_ENREF_50)] These conflicting results are not surprising due to the likely differences between protein binding behaviour in vivo versus in vitro studies. [[51](#_ENREF_51)]

Protein-unbound (free) drug is the pharmacologically active form of the drug as it is able to penetrate tissue more efficiently compared to protein-bound drug. [[43](#_ENREF_43), [44](#_ENREF_44)] Therefore the low protein binding at higher concentrations may be potentially of clinical significance in terms of azithromycin’s efficacy. Similarly low protein binding antiretroviral drugs (ARVs) were beneficial for tissue concentrations in female genital tissue [[32](#_ENREF_32)], however in contrast, this was not the case for rectal tissue concentrations with ARVs with high protein binding which achieved colorectal concentrations 2 to 12 fold greater exposure compared to female genital tissue. [[52](#_ENREF_52)] This may be related to the differences in active drug transporting systems between vaginal and colorectal tissue such as drug efflux transporters MRP2 and MRP4 in vaginal tissue and influx transporters OAT-1 in rectal tissue and greater P-glycoprotein efflux transporters in the vaginal tissue compared to rectal tissue. [[31](#_ENREF_31)] Although MRP4 was also found in rectal tissue, it was concentrated to lymphocytes rather than epithelial cells. The latter may also have implications for potentially reducing azithromycin levels in lymphocytes, a major vehicle for azithromycin’s delivery to the site of infections. This would then be balanced against the drug’s effects by active drug transport systems that result in its high intracellular accumulation. [[48](#_ENREF_48)]

Due to azithromycin’s low bioavailabilty and predominant elimination in faeces, approximately 47% of a 500mg dose is passed unchanged into the faeces. [[45](#_ENREF_45)] Drug in faeces therefore has the potential to be not only re-absorbed into the blood, [[53](#_ENREF_53)] but also for the drug to be delivered to rectal tissue and fluids (such as mucus) indirect contact. Studies with rectally applied tenofovir gel reported greater active drug concentrations in rectal tissue compared with oral dosing. [[41](#_ENREF_41)]

Positively charged (basic drugs) with low molecular weight have also been shown to bind electrostatically to the negatively charged components of mucus [[54](#_ENREF_54)] – so called ‘mucus trapping’. While mucus trapping is plausible for azithromycin, which chemically fits this description, [[55](#_ENREF_55), [56](#_ENREF_56)] binding to mucus has not been shown to date. Early studies in cervical mucus found azithromycin concentrations were above the MIC against chlamydia at 14 days following a single 1g dose [[28](#_ENREF_28)] and macrolides have been shown to concentrate in pulmonary epithelial lining fluid (ELF) [[57-59](#_ENREF_57)], with some authors suggesting pharmacokinetics in ELF were a better marker than those in the plasma. Mucus trapping of azithromycin in the rectum could therefore contribute to greater rectal tissue concentrations, especially when taken over multiple doses. [[54](#_ENREF_54)]

Given this, it is plausible that douching may have a deleterious effect on rectal azithromycin concentrations by reducing rectal tissue exposure to faeces and mucus containing azithromycin. Pre-sex rectal douching with non-isotonic fluids such as water is a common practice among MSM [[60](#_ENREF_60)] that can result in epithelial sloughing and damage to colonic tissue [[61](#_ENREF_61), [62](#_ENREF_62)] and reduce the production of mucus from damaged goblet cells [[61](#_ENREF_61)] - thereby reducing the potential for mucus trapping. This mechanism may possibly explain the association between enema use and rectal LGV infections, [[63](#_ENREF_63)] a 74% increased odds of reporting an STI in the past year [[60](#_ENREF_60)] and a 3.2 increased risk of acquiring hepatitis B. [[64](#_ENREF_64)] Similarly hyperosmolar water based lubricants have also been shown to cause damage to rectal tissue [[65-67](#_ENREF_65)] and potentially increase the risk of rectal STIs. [[68](#_ENREF_68)] The effects of rectal mucosal damage and increased STI infection risks can be likened to the breach of the mucosal protective barrier by genital ulcer disease in increasing HIV acquisition. [[11](#_ENREF_11)] Despite such studies however, a recent study in macaque showed no increased susceptibility to Simian HIV after application of a hyperosmolar lubricant despite evidence of rectal mucosal damage [[69](#_ENREF_69)]

Rectal samples/biopsies from pharmacokinetic studies of anti-retroviral drugs in rectal tissues sampled at 10cm [[39](#_ENREF_39)], 15cm [[41](#_ENREF_41), [42](#_ENREF_42)] and 10-30cm [[40](#_ENREF_40)] from the rectal margin or verge. The study collecting sample from 10-30cm showed no difference in infectability of HIV supporting that single site sampling was feasible.

Key pharmacokinetic parameters to investigate are summarised below in Table 2.

Table 2: Desired pharmacokinetics parameters

| Pharmacokinetic parameter | Implications/comment |
| --- | --- |
| Peak plasma concentrations (C_max_) and time to peak (t_max_) | Peak concentration following 500mg dose is reported as 0.3-0.4mcg/mL at 2-3 hours post dose [[46](#_ENREF_46)] and studies from a 3 or 5 day dosing of a 1.5g total dose reported minimum accumulation of drug on days 2-3 of a 3 day dosing regimen. [[70](#_ENREF_70)] |
| Peak tissue concentrations (C_max_) and time to peak (t_max_) | Peak tissue concentration is estimated at 24-48 hours post first dose with high concentrations persisting unchanged for over 8-days for one study [[71](#_ENREF_71)] |
| Tissue to plasma ratio (from above) | Extent of tissue penetration of the drug |
| Predictive pharmacodynamics parameter of azithromycin efficacy [[43](#_ENREF_43), [44](#_ENREF_44), [72](#_ENREF_72)] | |
| Peak plasma level to MIC ratio (C_max_/MIC). | Most relevant would be the maximum unbound (free) drug concentration (fCmax/MIC) [[73](#_ENREF_73)] as free (unbound) drug correlate with tissue penetration and effect of drugs and for bacteriostatic agents (eg macrolides) |
| 24 hour area under the curve (plasma) to MIC ratio (AUC_24_/MIC)* | - For pneumococcal infections a ratio of between 25-35 was predictive of efficacy for azithromycin and fluoroquinolones [[44](#_ENREF_44)]. - AUC/MIC ratio of greater than 5-100 was predictive of efficacy for respiratory pathogens using 2g extended release formulation of azithromycin [[74](#_ENREF_74)] - Free drug concentration-time curve (AUC) (f(AUC_24_)/MIC) may be more relevant. [[73](#_ENREF_73)] - 24h AUC/MIC correlates to the long in vivo post-antibiotic effect of the drug [[75](#_ENREF_75)] - with AUC_0-24_ as 2.6ug.hr/mL being previously reported. [[46](#_ENREF_46)] |
| Percentage of 24 hour time that unbound drug concentration exceeds the MIC (fT>MIC)* [[73](#_ENREF_73)] | - Free drug levels should be above the MIC for at least 40-50% of the dosing interval for bacteriological efficacy against susceptible respiratory organisms [[44](#_ENREF_44)]. - Free drug concentrations are important as they correlate with tissue penetration and effect of drugs and for bacteriostatic agents (eg macrolides) with the fT>MIC_90_ correlates with the antibacterial efficacy in animal models [[75](#_ENREF_75)] |

* AUC_24_/MIC would be more important than T>MIC for azithromycin given the marked persistent effects of the drug [[76](#_ENREF_76)]

Reference MIC is the MIC_90_^[[3]](#footnote-3)^ of 64ng/ml [[47](#_ENREF_47)]. Transition-point MIC (MIC_TP_)^[[4]](#footnote-4)^ [[77](#_ENREF_77)] and minimum chlamydicidal concentration (MCC)^[[5]](#footnote-5)^, especially the latter, has been found to be more consistent than the MIC for end-point assessment. Minimal bactericidal concentration (MBC) may also be considered with MBC defined as the drug concentration required to reduce infectious EB productionby >99% [[78](#_ENREF_78), [79](#_ENREF_79)]

Table 2: Azithromycin pharmacokinetic summary

| Dose (oral) | 500mg -  [[45](#_ENREF_45)] lukeFould97  250mg 12 hours apart | 500mg single dose [[80](#_ENREF_80)] Boonleang | 500mg single dose  [[81](#_ENREF_81)]  cooper | 500mg dose (day1)  [[82](#_ENREF_82)]  coates  (18-40yo) | 500mg dose (day1)  [[82](#_ENREF_82)]  (65-85yo) | 500mg single dose  [[83](#_ENREF_83)]  dunn | 500mg  single dose vs 2g ER  [[84](#_ENREF_84)] lucchi | | Single dose  [[85](#_ENREF_85)] liu  (30mg/kg IR vs  60mg/kg ER suspension) | Single dose  [[86](#_ENREF_86)] Baschiera2002  10 or 20mg/kg daily for 3 days as suspension |
| --- | --- | --- | --- | --- | --- | --- | --- | --- | --- | --- |
| Sample type | Ileostomy and serum | Serum  (for ‘generic drug’) | Serum (S) and inflamed blister (B) | 1.5g (500,250x4)  over 5 days.  Serum and urine | | Lung, serum, WBC (review) | Serum, ELF, AM and lung tissue  Data for Serum / ELF / Lung | | Acute otitis  media  doses:  IR:~420mg  ER:~786mg | Serum and tonsil tissue |
|  |  |  |  |  |  |  | 500mg single dose | 2g ER |  |  |
| Population | Ileostomy patients (n=12) | Healthy men (n=14) | Healthy men  (n=6) | 6 males and 6 females in each age group | | Children to adults  (n=N/A) | Lung cancer patients  (n=32 in each treatment arm) | | Children  (n=19) | Children undergoing surgery to remove tonsils (n=64) |
| Fasting (Y/N) | Y | Y | Y | Y | Y | - | - | - | Y | - |
| Cmax (ug/mL)  *ug/g for tissue | 0.21 | 0.425 | 0.45 (S)  0.13 (B) | 0.41 | 0.38 | 0.4-0.45 | Serum/ELF/Lung^+^  0.39/1.2/8.3 | Serum/ELF/Lung^+^  0.94/3.2/37.9 | 0.6 | Serum vs tonsil  10mg/kg: 0.13 vs 10.2  20mg/kg:~0.16 vs 16.6 |
| Tmax (h) | 2.5 | 1.5 | 2.5 (S)  3.25 (B) | 2.5 | 3.8 | 2.5 | 4/48/24 | 4/48/16 | 2-3 | Serum vs tonsil  12 vs 60 |
| AUC (ug.h/mL) | 1.27*(serum) | 4.34** | 1.9* (S)  1.52* (B) | 2.5*  (day1, serum) | 3.0*  (day1, serum) | 3.39*  (serum) | Serum  3.1* / 5** ^(AUC0-last)^  ELF/Lung^+^  2.3* / 130*  18.8** / 432**  AM:1674*/5804** | Serum  10* / 18.8**^(AUC0-last)^  ELF/Lung^+^  17.6* / 505*  131** / 1693**  AM:7028*/20403** | Serum:  IR: 3.96*  6.23**^(0-72)^  ER: 5.8*  9.8**^(0-72)^ | Serum vs tonsil ^(AUC0-204h)^  10mg/kg: 0.69** vs 61.1**  20mg/kg: 0.82** vs 98.1**  AUC units: mg x day1^-1^ (serum)  mg x day kg^-1^ (tissue) |
| T_1/2_ (hr) | - | 26.4 | - | - | - | 57 | - | - | - | - |
| Ke (h^-1^) | - | 0.03 | Ka=2.9/h (1.7-4.6) | - | - | - | - | - | - | - |
| Cl_Plasma_ | - | - | - | - | - | - | - | - | - | - |
| F (%) | 17.8  (62% dose in ileo fluid at 24hrs) | - | - | - | - | - | - | - | - | - |
| Vd (L/kg) | - | - | - | - | - | 23-31 |  |  | 518 (L, not L/kg) |  |
| AUC_0-24_/MIC_90_^ | 19.8 (serum) | 67.8** | 29.7 (S)  23.8 (B) | 39.1  (serum) | 46.9  (serum) | 53.0  (serum) | ELF/Lung  36* / 2031*  294**/ 6750** | ELF/Lung  275* / 7890*  2047**/26,453** | Serum  (* to **)  IR:62-97  ER:91-153 | - |
|  |  |  |  |  |  |  | At 24 hours, AUC_0-24_ 3.9-fold higher for ER than IR in lung tissue ie support ‘front end’ dosing | |  |  |

Cmax, peak plasma concentration; tmax, time- to Cmax; Ke, pharmacokinetic elimination constant; Ka: Absorption rate constant; Cl, plasmatic clearance; F, oral bioavailability; Vd, distribution volume. AUC, area under- (definite integral of-) the plasma concentration-versus-time curve; PMN: Polymorphonuclear; RBC=red blood cell; WBC=white blood cell; ELF=epithelial lining fluid; AM=alveolar macrophage; IR=immediate release; ER=extended release

* AUC0–24. ** AUC0–∞. ^using MIC_90_=64ng/ml (0.064ug/mL) for chlamydia trachomatis in theoretical scenario; +: assume mg/h/kg≈mcg/h/mL and mg/kg≈mcg/mL

Table 2: Azithromycin pharmacokinetic summary (continue)

| Dose (oral) | 500mg  [[87](#_ENREF_87)] curatolo  single dose (capsule) | 500mg  oral^[[6]](#footnote-6)^  [[29](#_ENREF_29)] fould90  500mg single dose,  3.5g over 5 days (1g day1),  2.75g over 9 days (500mg day1) | 500mg  [[88](#_ENREF_88)] fould91  250mg 12 hours apart | 500mg single dose  [[89](#_ENREF_89)] Baldwin | 500mg  [[90](#_ENREF_90)] krohn91  Single dose given 1,2,3, days pre surgery | 500mg  Single dose  [[91](#_ENREF_91)]  amsden | 1.5g  [[91](#_ENREF_91)]  over 3-5 days |
| --- | --- | --- | --- | --- | --- | --- | --- |
| Sample type | Fasting and fed study using fast dissolving formula | Tonsils, prostate, urological (testis, epididymis,vas deferens), gynaecological | Prostate and serum – data below for prostatic tissue.  *Other tissue include kidney, ureter, bladder, liver, bone, fat, muscle, adrenal gland* | Sputum (S), ELF, AM, bronchial mucosa (M) | Plasma, urine, peritoneal fluid & gynecological tissue. | Review paper.  Data from Parnham [[48](#_ENREF_48)] | |
| Population | Healthy adults  (n=12) | Healthy men  (n=10) | Men with prostate cancer (n=36) | Bronchoscopy patients (n=22) | Elective gynec surgical patients (n=20) | - | - |
| Fasting (Y/N) | Y and N. Results below (fasting/fed) | Y | - | - | Y | - | - |
| Cmax (ug/mL)    *ug/g for tissue | 0.48/0.25 | 0.41 | Time(hr)#/conc. (mcg.g)  11-18hr: 2.54  104-122hr: 0.74  137hr: 0.62 | Sputum/ELF/Mucosa/AM  1.56 / 2.18 / 3.89 / 23  *first sampling time=12 hrs post | Serum: 0.22mcg/mL  Gyne tissue: 1.44 mcg/g | 0.54 | 1.46 |
| Tmax (h) | 2/4 | - | - | 48 for all samples | Serum/tissue: 2/24 | - | - |
| AUC (ug.h/mL) | AUC 0-72h:  3.51/2.32 (serum)**  AUC 0-last:  3.76/2.32 (serum)** | -Serum (500mg):  2.36*, 3.08 ^(AUC 0-48h)^ ,  3.39 ^(AUC 0-72h)^  (Tissue 500mg dose: >2mg/L at 12-24rs and above MIC for >8 days | - | - | - | 11.2** | 13.1** |
| T_1/2_ (hr) | - | Serum=57, prostate=55, tonsil=77 | 60 | - | 67 | - | - |
| Ke (h^-1^) | - | - | Elimination constant 0.0116/hr | - | Depletion rate constant 0.0104/hr | - | - |
| Cl_Plasma_ | - | - |  | - |  | - | - |
| F (%) | - | - | - | - |  | - | - |
| Vd (L/kg) | - | - | - | - |  | 109.2 | 89.5 |
| AUC/MIC^ | Serum (AUC^0-72)^:  Fasted: 54.8**, Fed: 36.3**  Serum (AUC^0-last)^:  Fasted: 58.8**, Fed: 36.3** | Serum:  36.9 (500mg stat)  49.7 (3.5g/5d) ^(AUC 0-24h)^  19.1 (2.75g/9d) ^(AUC 0-12h)^  Tissue (12-24hr): >31.2 | Highest concentrations in urological tissue (9-51mcg/g in first 24 hours), and lowest in fat and muscle (~4mcg/g across all times) | - | * High gynecol. levels up to 96hours post dose | Serum:  175 | Serum:  204.7  ***AUC greater at day 3 vs day 5 (p=.06)*** |

Cmax, peak plasma concentration; tmax, time- to Cmax; Ke, pharmacokinetic elimination constant; Ka: Absorption rate constant; Cl, plasmatic clearance; F, oral bioavailability; Vd, distribution volume. AUC, area under- (definite integral of-) the plasma concentration-versus-time curve; PMN: Polymorphonuclear; RBC=red blood cell; WBC=white blood cell; ELF=epithelial lining fluid; AM=alveolar macrophage;

* AUC0–24. ** AUC0–∞. # 104-122hr=4.3-5.2 days and 136hr=5.7 days ^using MIC_90_=64ng/ml (0.064ug/mL) for chlamydia trachomatis in theoretical scenario;

Table 2: Azithromycin pharmacokinetic summary (continue)

| Dose (oral) | 500mg single dose  [[37](#_ENREF_37)]Harrison91 | 500mg single dose  [[93](#_ENREF_93)]Padwal2012 | 500mg single dose [[94](#_ENREF_94)] Idkaidek | 1g single dose  [[28](#_ENREF_28)] worm | 1g single dose  [[95](#_ENREF_95)] Vodstrcil | 1g single dose  [[35](#_ENREF_35)]Hoffler95 | 1g single dose  [[36](#_ENREF_36)] Bergan92 | 1g over 3 days  [[96](#_ENREF_96)] Ernst2000  (500mg,250mgx2) | 1g over 2 days  [[97](#_ENREF_97)]Margartis  caps |
| --- | --- | --- | --- | --- | --- | --- | --- | --- | --- |
| Sample type | Serum, gastric tissue, mucus and juice | Serum and gastric tissue | Serum and saliva | Cervical mucus  and serum | Cervical tissue (swab) and serum | Serum | Serum and lymph tissue | Serum and PMN. | Serum and sinus fluid |
| Population | Gastric cancer patients  (n=27) | Healthy females  (n=14) | Healthy adults  (n=3) | Chlamydia infected  women (n=20) | Healthy women  (n=10) | Healthy adults vs renal failure patients. Data for healthy adults  (n=12) | Healthy males  (n=14) | Healthy adults  (n=11) | Sinusitis patients  (n=16) |
| Fasting (Y/N) | Y | Y | Y | - | N | Y | Y | Y | Y |
| Cmax (ug/mL)  *ug/g for tissue | Tissue/mucus/juice  4.61/0.52/0.20  *1st sample at 24hours | Serum  0.36 | Saliva/Serum  2.3/0.13 | Serum: 24hr: 0.071ug/mL  (range: 0.024-0.126)  Mucus:  24hr: 2.67ug/g (0.57-9.51),  Day7: 1.26ug/g (0.39-5.65),  Day14:0.15ug/g (0.12-1.06) | Serum (av.)  3-4hr: 0.53 (0.10-1.02)  Cervical tissue:  Day2 (peak):0.92  (0.21-2.72)  Day9 (trough): 0.53  (0.10-1..02) | 1.07 | Serum/lymph  0.82/0.22 | Serum/PMN  0.27/57 | Serum/Sinus  0.61/0.87 |
| Tmax (h) | Tissue/mucus/juice  84/61/84 | 2.4 | Saliva/Serum  4.3/4.2 | - | Serum: 3-4 hours  Cervical tissue: day 2 | 1.8 | Serum/lymph  1.7/3.1 | 2.8/16.9 | Serum/Sinus  24/24 |
| AUC (ug.h/mL) | - | 2.07* | Saliva/Serum  5.6**/1.0** | - | - | **9.98** ^(AUC0-120h)^** | 7.9**/4.4** | 1.7*/6067** | - |
| T_1/2_ (hr) | - | - | - | - | - | 39.2 | Serum/lymph  44.2/50.8 | - | - |
| Ke (h^-1^) | - | - | - | - | - | - | - | - | - |
| Cl_Plasma_ | - | - | - | - | - | 522 (ml/min/1.73m2) | - | - | - |
| F (%) | - | - | - | - | - | - | - | - | - |
| Vd (L/kg) | - | - | - | - | - | 47 | - | - | - |
| AUC/MIC^ | - | 32.3 | 87.5/15.6 | - | -[[98](#_ENREF_98)] | 156 | 123/69  *After 120hrs, 63% of dose left in tissue | Serum  26.6*  (NB: * not **) | - |

Table 2: Azithromycin pharmacokinetic summary (continue)

| Dose (oral) | 1.5g over 3 days  [[99](#_ENREF_99)] matz13  (500mg dailyx3days) | 1.5g over 3 days [[100](#_ENREF_100)] Blandizzi99  (500mg dailyx3days) | 1.5g – single dose vs over 3 days [[101](#_ENREF_101)] Amsden2001 | 1.5g over 3 days vs 2g ER  [[102](#_ENREF_102)] Liu2007 | 1.5g over 3-5 days  [[70](#_ENREF_70)] Amsden99  (500mgx3 or 500mg day1 then 250mg day2-4) | 1.5g over 5 days  [[103](#_ENREF_103)]Krichhoff99 | 1.5g over 5 days oral  [[104](#_ENREF_104)] ballow  (500mg day1, 250mg dailyx4 days) |
| --- | --- | --- | --- | --- | --- | --- | --- |
| Sample type | Serum, extracell space of muscle and subcut tissue, intracell (WBC) [microdial probe used] | Serum, saliva, gingiva (healthy (H) and unhealthy (U)) | Serum, PMN, Monocyte/lymphocutes (ML) | Serum, PMN, MNL | Serum | Gastric tissue and juice | Serum, Urine, PMN and RBC and blister |
| Population | Men (n=6), **Respiratory and skin infections** | Chronic periodontitis/cyst (n=32, 50% male) | Healthy adults  (n=12) | Healthy adults  (n=24) | Healthy adults  (n=12) | Gastritis patients  (n=7; males) | Healthy men (n=14) |
| Fasting (Y/N) | Y | - | N | Y | Y | - | Y |
| Cmax (ug/mL)  *ug/g for tissue | Serum:  0.46 | Serum: 0.37  Saliva‡: 2.12  Gingiva‡(H/U): 6.3/11.6  *first sample at 12 hours | Serum/PMN/ML: single vs 3d  1.5/41/313 vs 0.5/31/165 | Serum/MNL/PMN: ER vs IR  0.73/116/146 vs 0.41/73/114 | - | **Tissue:**  Day2:7.5  Day5:9.7  Day9 (4 days post treatment cessation): 3.9  **Gastric juice:**  Not detected | 0.27 |
| Tmax (h) | 2.92 | All samples:  12 (first sampling time) | - | Serum/MNL/PMN: ER vs IR  3.5/8/12 vs 2/52/60 | - | - | - |
| AUC (ug.h/mL) | Serum: 3.05* / 17.4**[[46](#_ENREF_46)]  Muscle: 0.308*  WBC: 436*  **(AUC/MIC: Peaks at day 3)** | - | Serum/PMN/ML: single vs 3d  13/6447/20461** vs 11/5128/15706**  Serum=**  PMN/ML= AUC0-240** | Serum/MNL/PMN: ER vs IR*  8/1790/2080 vs 3/647/704  Serum/MNL/PMN: ER vs IR** ^(AUC0-120h)^  15/4710/10000 vs 14/3890/7830 | 3 vs 5 day  19.4** vs 15.9** | - | Inflam blister:  7.54*  Non-inflam blister: 4.53*  Serum (av): 3.64* |
| T_1/2_ (hr) | 71.8 [[46](#_ENREF_46)] | - | - | - | 3 vs 5 day  65.9 vs 66.1 | - | 78.6 |
| Ke (h^-1^) | - | - | - | 164mg/h (zero-order absorption rate, serum) | - | - | Ka=0.619 (mean)  vs 0.452 when F=40% [[105](#_ENREF_105)] |
| Cl_Plasma_ | - | - | Serum: single vs 3 days  125 vs 153 (L/h) | - | - | - | - |
| F (%) | - | - |  | - | - | - | - |
| Vd (L/kg) | - | - | Serum: single vs 3 days  90 vs 109 | 122L/h (IR serum) | - | - | - |
| AUC/MIC^ | 4.8 (muscle)  6813 (WBC)  47.7 (serum)  NB: data similar to review data [[46](#_ENREF_46), [106](#_ENREF_106)] – see below | *‡(mg/kg) highest in saliva and H at 4.5days and 6.5days respectively (p<0.01)* | Serum: single vs 3 day  205 vs 175**  ***AUC similar between single and 3 day course*** | Serum: ER vs IR  123 vs 42*  231 vs 214**  ***AUC for 2g ER similar to 1.5g over 3 days. AUC 3-fold higher for ER vs IR at day1 ie support ‘front end’ loading dose.*** | Serum  303** vs 248**  ***3 day regimen similar to 5 day regimen*** | -  *possible interaction by co-administered acid lowering drug (pantoprazole) | 118 (inflamed)  71 (non-inflamed)  57 (serum) |

Table 2: Azithromycin pharmacokinetic summary (continue)

| Dose (oral) | 1.5g vs 3g over 3 days  [[107](#_ENREF_107)] Di Paolo  500mg or 1g x 3days  (capsule) | 1.5g vs 3g over 3 days  [[108](#_ENREF_108)] danesi  500mg or 1g x 3days (capsule) | 2g single  dose as powder suspension ER vs IR [[109](#_ENREF_109)] – chandra | 2g single  dose as powder suspension ER vs 1.5g over 3 days (500mgx3) [[110](#_ENREF_110)] ehnhage | 3.5g over 5 days and 2.75g over 9 days  [[29](#_ENREF_29)] foulds90 | 17.5g over 35 days  [[111](#_ENREF_111)]Wilms  500mg daily for >35 days | 4.5g over23 days  [[112](#_ENREF_112)]  (1.5g over 3days) x 3; each separated by 7days |
| --- | --- | --- | --- | --- | --- | --- | --- |
| Sample type | Serum, lung (L) and  brochial washings (BW) | Serum, lung (L) and  brochial washings (BW) | Serum. Also data on high fat meals, standard meal and with antacids | Serum, sinus fluid | Serum, urogenital, tonsil, prostate, lung, kidney, muscle, fat,bone | Plasma (P), blood (B) and neutrophils (N) | Serum and gastric tissue |
| Population | Lung resection patients  (n=28 in each treatment group) | Lung resection patients  (n=24 in each group) | Healthy males and females  (n=16 cross-over study) | Adults with acute bacterial sinusitis (n=5 for 2g ER and n=4 for 1.5g over 3 days) | Surgical  (n=12 for 3.5g and n=20 for 2.75g dose regimen) | Cystic fibrosis patients (n=8) | Duodenal ulcer patients  (n=20) |
| Fasting (Y/N) | Y | Y | Y | - | - | - | - |
| Cmax (ug/mL)  *ug/g for tissue | 1.5g vs 3g  Serum: 0.26 vs 0.32  BW: 0.72 vs 1.41  Lung: 9.13 vs 17.85 | 1.5g vs 3g:  Serum: 0.18 vs 0.32  BW: 0.83 vs 1.5  Lung: 8.9 vs 18.6 | ER vs IR  0.85 vs 2.1 | 2g ER vs 1.5g  Serum: 1.09 vs 0.28  Sinus fluid: 3.2 vs 1.1 | Serum:  0.2-0.21 (2.75g)  0.41-0.62 (3.5g) | P/B/N  0.67/2.01/1.44 | Serum/tissue  0.32/21.6  *1^st^ sample at day4 |
| Tmax (h) | 1.5g vs 3g  Serum: 12 vs 12  BW: 12 vs 120  Lung: 60 vs 60 | Serum: 12 hours  BW: 12 hours  Lung: 60 hours | 4.1 vs 1.6 | 2g ER vs 1.5g  Serum: 4 vs 2.5  Sinus fluid: 24 vs 48 | - | P/B/N  3/3/4 | Day 4 |
| AUC (ug.h/mL) | 1.5g vs 3g ^(AUC0-204)^  Serum: 20.48 vs 25.6**  BW: 60.6 vs 135.1**  Lung: 1318 vs 2502** | 1.5g vs 3g ^(AUC0-204hr)^  Serum: 11.62 vs 19.83**  BW: 70.3 vs 140**  L: 1245 vs 2514** | Serum ^(AUC0-96h)^  16.8 vs 19.5 | 2g ER vs 1.5g  Serum: 6.7* vs 1.7*  Sinus fluid: 23.4* vs 7.9* | Serum ^(AUC^**^0-12)^**  0.8-1.22 (2.75g)  1.77-3.18 (3.5g) | P/B/N  5.3*/27.8*/18.5* | - |
| T_1/2_ (hr) | 1.5g vs 3g  Serum: 65.6 vs 62.6  BW: 74.3 vs 70.5  Lung: 132.9 vs 133.3 | Serum: 38.5 vs 44.6 | - | - | prostate=55, tonsils=77 | P/B/N  102/178/289  (extended T_1/2_) | - |
| Ke (h^-1^) | - |  | - | - | - | - | - |
| Cl_Plasma_ | - | Serum: 43.0 vs 50.5 L/hr | - | - | - | - | - |
| F (%) | - |  | - | - | - | - | - |
| Vd (L/kg) | - | 2387 vs 3247 | - | - | - | - | - |
| AUC/MIC^ | 1.5g vs 3g  Serum: 320 vs 400**  BW: 947 vs 2111**  L: 20,594 vs 39,094** | 1.5g vs 3g  Serum: 182 vs 310**  BW: 1,098 vs 2186**  L: 19,459 vs 39,284** | Serum(AUC_96h_):  262 vs 305 | 2g ER vs 1.5g  Serum: 104.7* vs 26.6*  Sinus fluid: 365.6* vs 123.4* | Serum **(AUC0-12 hrs)**  19.1 (2.75g)  49.7 (3.5g) | P/B/N  83/434/289  *chronic use results in higher accumulation in neutrophils vs short term use | -  *possible interaction by co-administered acid lowering drug (omeprazole) |

Table 2: Azithromycin pharmacokinetic summary (continue)

| Dose/route providing data | Pfizer product information | | | | | | | | |
| --- | --- | --- | --- | --- | --- | --- | --- | --- | --- |
|  | 500mg oral  [[46](#_ENREF_46)] Canada  -250mg 12 hours apart | 1g oral  [[113](#_ENREF_113)] AUS – single dose  **DELETE**  SEE ABOVE RENAL POPN | 1.2g oral  [[46](#_ENREF_46)] – single dose | Powder for suspension [[46](#_ENREF_46)] Canada | | | Powder for suspension  [[114](#_ENREF_114)] USA | 2g extended release (ER) suspension single dose vs  1.5g over 3-5days [[114](#_ENREF_114)] USA | |
|  |  |  |  | 10mg/kg (day 1) then 5mg/kg (day2-5):  *Data for age 1-5yo vs 5-15yo* | 60mg/kg over 3-5 days | | 60mg/kg single dose  (max 2g) | 2g ER single dose | 1.5g (Serum)  (3days vs 5days) |
|  |  |  |  |  | Over 3 days  (20mg/kg x 3 days) | Over 5 days  (12mg/kg x 5 days) |  |  |  |
| Sample type | Serum, tonsil | Serum | Serum | Serum | Serum | Serum | Serum | Serum | Serum |
| Population | - | - | Adult  (n=12) | Children  (n=N/A) | Children with pharyngitis/ tonsilitis  (n=34) | Children with  pharyngitis/ tonsilitis  (n=31) | Children aged 3mth to 16yo (n=36; fasting) and 18mth to 8 years (n=7; non-fast);  data fasting vs non-fasting | Healthy adults  (n=41) | Healthy adults  (n=12 in both 3 day and 5 day regimen) |
| Fasting (Y/N) | N | - | Y | - | N | N | Y (n=36)/N (n=7) | Y | - |
| Cmax (ug/mL)  *u g/g for tissue | 0.3-0.4 | 1.0 | 0.66 | 0.216/0.383 | 1.05 | 0.534 | 1.27 / 1.41 | 0.821 | 0.441 / 0.434 |
| Tmax (h) | 2.3 |  | 2.5 | 1.9/2.4 | 3 | 2.2 | 3 / 3 | 5 | 2.5 / 2.5 |
| AUC (ug.h/mL) | Serum  2.6* and 3.7 (AUC_0-48_) Tonsil  4.5 ug/g (9-18 hrs)  0.93ug/g(180 hrs or 7.5 days) | 8.8  (AUC_0-120hr_) | Serum  6.8** | Serum  1.82* / 3.11* | Serum  7.92* | Serum  3.94* | 13.1* / 7.4*  25.2** / 18.9** | Serum  8.62*  20.0** | Serum (3 vs 5 day)  3 day: 2.58*/ 17.4**  5 day: 2.6* / 14.9** |
| T_1/2_ (hr) | 68 |  | 40 | - | - | - | - | 58.8 | 71.8/68.9 |
| Ke (h^-1^) | - | - | - | - | - | - | - |  | 0.0101 [[46](#_ENREF_46)] |
| Cl_Plasma_ | 630 mL/min | 2.3mL/min/kg | - | - | - | - | - |  |  |
| F (%) | 37 | - | - | - | - | - | - | 83 | - |
| Vd (L/kg) | 31.1 | - | - | - | - | - | - |  |  |
| AUC/MIC^ | Serum:  40.6* and 58 (AUC_0-48_)  Tonsil: 70 (9-18 hrs)  14.5 (180 hrs or 7.5 days) | Serum:  138 | Serum:  106 | Serum:  28.5/48.6 | Serum:  123.8 | Serum:  61.6 | Serum:  204* / 116*  393** / 295** | Serum:  135*  313** | Serum: (3 vs 5 day)  3 day: 40.3* / 272**  5 day: 40.6* / 232** |
|  |  |  |  |  |  |  |  | ***NB: AUC of 2g ER similar to 1.5g over 3-5 days***  NB: data similar to Harrison et al review data [[106](#_ENREF_106)] | |

# APPENDIX 2 - Pharmacokinetic of azithromycin in vaginal tissue

Presented at the 13th International Symposium on Human Chlamydial Infections – California June 2014

POST-TREATMENT DETECTION OF AZITHROMYCIN IN HIGH-VAGINAL SWABS USING LIQUID CHROMATOGRAPHY AND TANDEM MASS SPECTROMETRY (LC-MS/MS)

LA Vodstrcil^1,2^, T Rupasinghe^3^, D Tull^3^, K Worthington^1,4^, M Chen^1,4^, WM Huston^5^, CK Fairley^4^, M McConville^3^, SN Tabrizi^2, 6, 7^, JS Hocking^1^.

^1^Centre for Epidemiology and Biostatistics, Melbourne School of Population and Global Health, University of Melbourne, Parkville, Australia; ^2^Murdoch Children’s Research Institute, Parkville, Australia; ^3^Metabolomics Australia, Bio21 Institute, University of Melbourne, Parkville, Australia; ^4^Melbourne Sexual Health Centre, Carlton, Australia; ^5^Faculty of Health - Biomedical Sciences, Institute of Health and Biomedical Innovation, Queensland University of Technology, Brisbane, Australia; ^6^Department of Microbiology and Infectious Diseases, The Royal Women’s Hospital, Parkville, Victoria, Australia; ^7^Department of Obstetrics and Gynaecology, University of Melbourne, The Royal Women’s Hospital Parkville, Victoria, Australia

Introduction

Azithromycin is rapidly absorbed from the gastrointestinal tract following oral administration, with serum concentrations of azithromycin at highest levels 3-4 hours later. Azithromycin is then delivered into intracellular tissue resulting in high tissue concentrations. In tissues, azithromycin is slowly released, resulting in a long terminal phase elimination half-life [[115](#_ENREF_115), [116](#_ENREF_116)], making it suitable to be used as a single dose treatment. A 1 gram single dose of azithromycin has been reported to be as effective as 7-day regimens of other drugs, such as doxycycline, for the treatment of genital chlamydia and is the most widely recommended first line treatment for this infection [[117-120](#_ENREF_117)].

Recently however, the effectiveness of a single-dose of 1g azithromycin is under debate [[121-125](#_ENREF_121)]. Repeat infection with chlamydia is common following treatment with 1 gram azithromycin. While most repeat infections are generally re-infections, emerging evidence suggests that treatment failure with azithromycin may account for a substantial proportion. Studies have reported treatment failures of about 8% following 1 gram azithromycin in women in whom the risk of re-infection has been ruled out [[126](#_ENREF_126), [127](#_ENREF_127)].

Although azithromycin levels are thought to remain well above the reported minimum inhibitory concentration (MIC) for chlamydia between 10 to 14 days post-treatment with 1g dose [[128](#_ENREF_128), [129](#_ENREF_129)], there are limited data in the literature to support this. The aim of the current study was to quantify the concentration of azithromycin using liquid chromatography and tandem mass spectrometry (LC-MS/MS) [[130-132](#_ENREF_130)] in high-vaginal material (cells and mucus) self-collected by 10 women over 9 days after treatment with 1g of azithromycin.

Methods

Ethical approval for this study was granted by the Alfred Hospital Ethics Committee and The University of Melbourne Central Human Research Ethics Committee. Ten healthy women were recruited from the University of Melbourne and the Melbourne Sexual Health Centre, Victoria Australia. Demographics recorded by each participant included age, height, weight, any current medication and any current vaginal gel use. Women were then given instruction on self-collecting a high-vaginal swab (baseline). After collecting their baseline swab, all participants took a 1g dose of azithromycin.

The ten women were instructed to self-collect a high-vaginal swab every day for 9 further days. All swabs were analysed using liquid-chromatography tandem mass spectrometry (LC-MS/MS). A blood sample was also collected from all participants 3-4 hours after their initial azithromycin dose to determine plasma concentrations of azithromycin. This was also analysed using LC-MS/MS.

*LC-MS/MS*

Specimen preparation

All high-vaginal swabs were immediately agitated in 1ml of cold 100% methanol (MeOH) and then placed at -80ºC for up to two weeks before analysis. Leucine enkaphlin obtained from Sigma Aldrich (Australia) with 99.9% purity was used as the internal standard (IS) for sample preparation. For extraction, 1ml of chloroform containing the IS at a concentration of 1µg/mL was added to each sample and vortexed for 1 min. Samples were then agitated for 30 mins at 30ºC on a mechanical shaker and centrifuged at 13000rpm for 15 mins. The organic layer was then dried under a gentle stream of nitrogen at 40ºC. The residue was reconstituted in 100µl of 100% MeOH. Absolute quantitation of azithromycin present in the specimens was carried out using azithromycin standards (see below). Lipid concentration was also determined to normalise for differences in swab collection both within and between participants.

Preparation of azithromycin standards

A 500mg azithromycin tablet weighing 942mg was powdered using a motor and pestle, and then 559mg of powder dissolved in 29.6mL of 100% MeOH to prepare an equivalent concentration of 10mg/mL stock azithromycin solution. This was then filtered through MILLEX^R^GP 0.22µm PES membrane (MILLIPORE, Corristwohill co, Cork, Ireland) and used as a pure standard.

Two additional women who did not receive azithromycin also self-collected a total of 10 swabs using the same methodology described above. This was done to collect vaginal cellular material to be used as the sample matrix. The sample matrix is required for preparation of the standard curve as it contains similar vaginal cellular material as for the other 10 women, but without any azithromycin. Nine of these samples from the two women were spiked with azithromycin concentrations ranging from 0.1-1000ηg/mL, and one sample was left blank as a negative control. Calibration curves were generated by assaying these samples. The linearity of each calibration curve was determined by plotting the nominal concentration of azithromycin to the peak area ratio of azithromycin normalised to the lipid content of each tissue sample.

Instrument and conditions

The system used was a triple quad (QQQ) Mass Spectrometer (MS) (Agilent 6460 LC-MS/Agilent 6490 LC-MS) by multiple reaction monitoring (MRM) in positive mode. An Agilent Porshell 120 SB-C18 (2.7µm) 2.1 x 100mm column was used, maintained at room temperature. The isocratic mobile phase comprised 20 mM Ammonium Acetate in (1:1/ (v/v)) Acetonitrile: MeOH, delivered at a flow rate of 0.5 mL/min and a run time of 12 minutes. Mass spectrometry detection was carried out by MRM transition of *m/z* 749.0 🡪 591.6 and 556 🡪 397 for azithromycin and IS respectively.

Extracted lipids were separated by injecting 5µL aliquots of the prepared sample onto a 50mm × 2.1mm × 2.7µm Ascentis Express RP-Amide column (Supelco) using an Agilent LC 1200. Samples for lipid detection were eluted at 0.2mLmin-1 over a 5 min gradient of water/ MeOH/ tetrahydrofuran (50:20:30, v/v/v) to water/ MeOH/ tetrahydrofuran (5:20:75, v/v/v), with the final buffer held for 3 mins. Lipids were analysed by electrospray ionisation-mass spectrometry (ESI-MS) using an Agilent Triple Quad 6460. Mass spectrometry detection was carried out by MRM transition of m/z 760 🡪 184 to quantify the lipid species of PC(34:1). The capillary voltage, fragmentor voltage, and collision energy were 4000 V, 140 – 380 V, and 15–60 V, respectively. In all cases, the collision gas was nitrogen at 7 Lmin-1. For all samples and standards, LC-MS data was processed using the Agilent MassHunter quantitative software (version 5).

Results

Azithromycin was detected at varying concentrations in all 10 women in all post-treatment samples. The highest average normalised azithromycin concentration of 916ng/ml (range= 213-2722ηg/mL) was detected on day 2 post-treatment (Figure 1). The lowest average azithromycin concentration was 119ηg/mL (range= 51-396ηg/mL), 9 days post-treatment. The average concentration of azithromycin detected in blood samples was 533ηg/mL (range= 104-1019ηg/mL). In 7/10 women azithromycin concentrations remained above 64ηg/mL, the hypothesised mean inhibitory concentration (MIC) of azithromycin for chlamydia [[133](#_ENREF_133)], for the entire 9 days; in 3/10 women, the levels dropped below this hypothesized mean MIC from day 8 or 9. Leucine enkaphalin levels remained consistent across samples and runs indicating uniform specimen preparation.

Figure 1. Mean concentration of azithromycin normalised to lipid concentration for 9-days following a 1g dose in 10 participants.

Discussion

We have developed and validated an assay for detecting the azithromycin concentration in self-collected high-vaginal samples using LC-MS/MS. Azithromycin concentrations remained above the reported (hypothetical) MIC of 64ηg/mL [[133](#_ENREF_133)] for up to 8 days post-treatment in high-vaginal swabs from 10 healthy women. This assay is being used to determine whether azithromycin is being absorbed to the site of infection in an ongoing cohort study of women infected with cervical chlamydia infection that aims to estimate the risk of treatment failure following 1 gram azithromycin [[134](#_ENREF_134)]. If cases of possible azithromycin treatment failure are identified in this cohort study, our assay will be used to determine whether treatment failure occurred because the antibiotic did not make it to the site of infection.

# REFERENCES

1. World Health Organisation. Global incidence and prevalence of selected curable sexually transmitted infections – 2008. **2008**; accessed January 2013.

2. National Notifiable Diseases Surveillance System. Number of notifications of Chlamydial infections, Australia, by age group and sex. **2014**; <http://www9.health.gov.au/cda/source/cda-index.cfm:> accessed February 2015.

3. Public Health England (PHE). Sexually Transmitted Infections Annual Data. Available at: https://[www.gov.uk/government/statistics/sexually-transmitted-infections-stis-annual-data-tables](http://www.gov.uk/government/statistics/sexually-transmitted-infections-stis-annual-data-tables). Accessed 28/07/2014.

4. Statens Serum Institut. EPI-NEWS. Chlamydia 2012 **2013**; 36(28/07/2104): <http://www.ssi.dk/English/News/EPI-NEWS/2013/No%36%20-%202013.aspx>.

5. European Centre for Disease Prevention and Control (ECDC). Reporting on 2011 surveillance data and 2012 epidemic intelligence data. **Annual Epidemiological Report 2013.**; <http://www.ecdc.europa.eu/en/publications/Publications/Annual-Epidemiological-Report-2013.pdf>.

6. Lewis D, Newton D, Guy R, et al. The prevalence of Chlamydia trachomatis infection in Australia: a systematic review and meta-analysis. BMC Infectious Diseases **2012**; 12(1): 113.

7. Annan N, Sullivan A, Nori A, et al. Rectal chlamydia - a reservoir of undiagnosed infection in men who have sex with men. Sexually Transmitted Infections **2009**; 85: 176 - 9.

8. Munson E, Wenten D, Phipps P, et al. Retrospective assessment of transcription-mediated amplification-based screening for Trichomonas vaginalis in male sexually transmitted infection clinic patients. Journal of Clinical Microbiology **2013**; 51(6): 1855-60.

9. Kent CK, Chaw JK, Wong W, et al. Prevalence of rectal, urethral, and pharyngeal Chlamydia and Gonorrhea detected in 2 clinical settings among men who have sex with men: San Francisco, California, 2003. Clinical Infectious Diseases **2005**; 41(1): 67-74.

10. Dudareva-Vizule S, Haar K, Sailer A, et al. Prevalence of pharyngeal and rectal Chlamydia trachomatis and Neisseria gonorrhoeae infections among men who have sex with men in Germany. Sexually Transmitted Infections **2014**; 90(1): 46-51.

11. Fleming DT, Wasserheit JN. From epidemiological synergy to public health policy and practice: the contribution of other sexually transmitted diseases to sexual transmission of HIV infection. Sexually Transmitted Infections **1999**; 75(1): 3-17.

12. Rottingen JA, Cameron DW, Garnett GP. A systematic review of the epidemiologic interactions between classic sexually transmitted diseases and HIV: how much really is known? Sexually Transmitted Diseases **2001**; 28(10): 579-97.

13. Ward H, Ronn M. Contribution of sexually transmitted infections to the sexual transmission of HIV. Curr Opin HIV AIDS **2010**; 5(4): 305-10.

14. Zuckerman RA, Whittington WLH, Celum CL, et al. Higher Concentration of HIV RNA in Rectal Mucosa Secretions than in Blood and Seminal Plasma, among Men Who Have Sex with Men, Independent of Antiretroviral Therapy. Journal of Infectious Diseases **2004**; 190(1): 156-61.

15. Pathela P, Braunstein SL, Blank S, Schillinger JA. HIV Incidence Among Men With and Those Without Sexually Transmitted Rectal Infections: Estimates From Matching Against an HIV Case Registry. Clinical Infectious Diseases **2013**; 57(8): 1203-9.

16. Jones C, Kuldanek K, Phekoo K, et al. Strategies to guide HIV prevention approaches: Correlation of sexually transmitted infections and sexual behaviour with risk of HIV infection. In: 19th Annual Conference of the British HIV Association, BHIVA 2013, 2013.

17. Bernstein KT, Marcus JL, Nieri G, Philip SS, Klausner JD. Rectal gonorrhea and chlamydia reinfection is associated with increased risk of HIV seroconversion. Journal of Acquired Immune Deficiency Syndromes: JAIDS **2010**; 53(4): 537-43.

18. Jamani S, Gulholm T, Poynten IM, Templeton DJ. Timing and frequency of chlamydia and gonorrhoea testing in a cross-sectional study of HIV postexposure prophylaxis recipients. Sexually Transmitted Infections **2013**; 89(7): 604-6.

19. Centers for Disease Control and Prevention. Sexually Transmitted Diseases Treatment Guidelines, 2010. MMWR - Morbidity & Mortality Weekly Report **2010**; 59(No.RR-12): 1-116.

20. Bachmann L, Stephens J, Richey C, Hook E. Measured versus self-reported compliance with doxycycline therapy for chlamydia-associated syndromes - high therapeutic success rates despite poor compliance. Sex Transm Dis **1999**; 26(5): 272 - 8.

21. Jordan SJ, Geisler WM. Azithromycin for Rectal Chlamydia: Is it Time to Leave Azithromycin on the Shelf?...Not Yet. Sexually Transmitted Diseases **2014**; 41(2): 86-8.

22. Steedman NM, McMillan A. Treatment of asymptomatic rectal Chlamydia trachomatis: is single-dose azithromycin effective? International Journal of STD & AIDS **2009**; 20(1): 16-8.

23. Drummond F, Ryder N, Wand H, et al. Is azithromycin adequate treatment for asymptomatic rectal chlamydia? International Journal of STD & AIDS **2011**; 22(8): 478-80.

24. Hathorn E, Opie C, Goold P. What is the appropriate treatment for the management of rectal Chlamydia trachomatis in men and women? Sexually Transmitted Infections **2012**; 88(5): 352-4.

25. Khosropour Christine M., Dombrowski Julia C., Barbee LA., Manhart LE., Golden MR. Comparing azithromycin and doxycycline for the treatment of rectal Chlamydial Infection: A retrospective cohort study. Sexually Transmitted Diseases **2014**; 41(2): 79-85

26. Kong FYS, Tabrizi SN, Law M, et al. Azithromycin versus doxycycline for the treatment of genital chlamydia infection – a meta-analysis of randomised controlled trials. Clinical Infectious Diseases **2014**; 59(2): 193-205.

27. Kong FYS, Tabrizi SN, Fairley CK, et al. The efficacy of azithromycin and doxycycline for the treatment of rectal chlamydia infection: a systematic review and meta-analysis. Journal of Antimicrobial Chemotherapy **2015**.

28. Worm AM, Osterlind A. Azithromycin levels in cervical mucus and plasma after a single 1.0g oral dose for chlamydial cervicitis. Genitourinary Medicine **1995**; 71(4): 244-6.

29. Foulds G, Shepard RM, Johnson RB. The pharmacokinetics of azithromycin in human serum and tissues. Journal of Antimicrobial Chemotherapy **1990**; 25 Suppl A: 73-82.

30. Heiligenberg M, Lutter R, Pajkrt D, et al. Effect of HIV and Chlamydia Infection on Rectal Inflammation and Cytokine Concentrations in Men Who Have Sex with Men. Clinical and Vaccine Immunology **2013**; 20(10): 1517-23.

31. Nicol MR, Fedoriw Y, Mathews M, et al. Expression of six drug transporters in vaginal, cervical, and colorectal tissues: Implications for drug disposition in HIV prevention. The Journal of Clinical Pharmacology **2014**; 54(5): 574-83.

32. Trezza C, Kashuba AM. Pharmacokinetics of Antiretrovirals in Genital Secretions and Anatomic Sites of HIV Transmission: Implications for HIV Prevention. Clin Pharmacokinet **2014**; 53(7): 611-24.

33. Anderson PL, Kiser JJ, Gardner EM, Rower JE, Meditz A, Grant RM. Pharmacological considerations for tenofovir and emtricitabine to prevent HIV infection. Journal of Antimicrobial Chemotherapy **2011**; 66(2): 240-50.

34. World Health Organisation. Critically Important Antimicrobials for Human Medicine **2011**.

35. Hoffler D, Koeppe P, Paeske B. Pharmacokinetics of azithromycin in normal and impaired renal function. Infection **1995**; 23(6): 356-61.

36. Bergan T, Jorgensen NP, Olszewski W, Zhang Y. Azithromycin pharmacokinetics and penetration to lymph. Scandinavian Journal of Infectious Diseases, Supplement **1992**; 23(83): 15-21.

37. Harrison JD, Jones JA, Morris DL. Azithromycin levels in plasma and gastric tissue, juice and mucus. Eur J Clin Microbiol Infect Dis **1991**; 10(10): 862-4.

38. Kirchhoff RM, Laufen H, Schacke G, Kirchhoff G, Gallo E. Determination of azithromycin in gastric biopsy samples. International Journal of Clinical Pharmacology and Therapeutics **1999**; 37(7): 361-4.

39. Patterson KB, Prince HA, Kraft E, et al. Penetration of tenofovir and emtricitabine in mucosal tissues: implications for prevention of HIV-1 transmission. Science translational medicine **2011**; 3(112): 112re4-re4.

40. Anton PA, Saunders T, Elliott J, et al. First Phase 1 Double-Blind, Placebo-Controlled, Randomized Rectal Microbicide Trial Using UC781 Gel with a Novel Index of Ex Vivo Efficacy. PloS one **2011**; 6(9): e23243.

41. Anton PA, Cranston RD, Kashuba A, et al. RMP-02/MTN-006: a phase 1 rectal safety, acceptability, pharmacokinetic, and pharmacodynamic study of tenofovir 1% gel compared with oral tenofovir disoproxil fumarate. AIDS research and human retroviruses **2012**; 28(11): 1412-21.

42. Patterson KB, Prince HA, Stevens T, et al. Differential penetration of raltegravir throughout gastrointestinal tissue: implications for eradication and cure. Aids **2013**; 27(9): 1413-9 10.097/QAD.0b013e32835f2b49.

43. Andes D. Pharmacokinetic and pharmacodynamic properties of antimicrobials in the therapy of respiratory tract infections. Current Opinion in Infectious Diseases **2001**; 14(2): 165-72.

44. Craig WA. Does the Dose Matter? Clinical Infectious Diseases **2001**; 33(Supplement 3): S233-S7.

45. Luke DR, Foulds G. Disposition of oral azithromycin in humans. Clin Pharmacol Ther **1997**; 61(6): 641-8.

46. Pfizer Canada. Zithromax product information. Canada, **2013**

47. Suchland RJ, Geisler WM, Stamm WE. Methodologies and cell lines used for antimicrobial susceptibility testing of Chlamydia spp. Antimicrobial Agents & Chemotherapy **2003**; 47(2): 636-42.

48. Parnham MJ, Haber VE, Giamarellos-Bourboulis EJ, Perletti G, Verleden GM, Vos R. Azithromycin: Mechanisms of action and their relevance for clinical applications. Pharmacology & Therapeutics **2014**; 143(2): 225-45.

49. Birkett DJ. Pharmacokinetics made easy 9: Non-linear pharmacokinetics. Australian Prescriber **1994**; 36-8: 36-8.

50. Gastearena I, Dios-Vieitez M, Terraz M, Domingo S, Fos D. Determination of the alpha1-acid glycoprotein binding of azithromycin in vitro by equilibrium dialysis. Journal of chemotherapy (Florence, Italy) **1995**; 7: 26-8.

51. Kremer JM, Wilting J, Janssen LH. Drug binding to human alpha-1-acid glycoprotein in health and disease. Pharmacological Reviews **1988**; 40(1): 1-47.

52. Brown KC, Patterson KB, Jennings SH, et al. Single- and multiple-dose pharmacokinetics of darunavir plus ritonavir and etravirine in semen and rectal tissue of HIV-negative men. J Acquir Immune Defic Syndr **2012**; 61(2): 138-44.

53. Bergogne-Bérézin E, Bryskier A. The suppository form of antibiotic administration: pharmacokinetics and clinical application. Journal of Antimicrobial Chemotherapy **1999**; 43(2): 177-85.

54. Khanvilkar K, Donovan MD, Flanagan DR. Drug transfer through mucus. Advanced Drug Delivery Reviews **2001**; 48(2–3): 173-93.

55. Farmer S, Li Z, Hancock REW. Influence of outer membrane mutations on susceptibility of Escherichia coli to the dibasic macrolide azithromycin. Journal of Antimicrobial Chemotherapy **1992**; 29(1): 27-33.

56. Montenez J-P, Van Bambeke F, Piret J, et al. Interaction of the macrolide azithromycin with phospholipids. II. Biophysical and computer-aided conformational studies. European journal of pharmacology **1996**; 314(1): 215-27.

57. Drusano GL. Infection Site Concentrations: Their Therapeutic Importance and the Macrolide and Macrolide-Like Class of Antibiotics. Pharmacotherapy: The Journal of Human Pharmacology and Drug Therapy **2005**; 25(12P2): 150S-8S.

58. Rodvold KA, Danziger LH, Gotfried MH. Steady-State Plasma and Bronchopulmonary Concentrations of Intravenous Levofloxacin and Azithromycin in Healthy Adults. Antimicrobial Agents And Chemotherapy **2003**; 47(8): 2450-7.

59. Rodvold KA, Gotfried MH, Danziger LH, Servi RJ. Intrapulmonary steady-state concentrations of clarithromycin and azithromycin in healthy adult volunteers. Antimicrobial Agents And Chemotherapy **1997**; 41(6): 1399-402.

60. Javanbakht M, Stahlman S, Pickett J, LeBlanc M-A, Gorbach P. Prevalence and types of rectal douches used for anal intercourse: results from an international survey. BMC Infectious Diseases **2014**; 14(1): 95.

61. Schmelzer M, Schiller LR, Meyer R, Rugari SM, Case P. Safety and effectiveness of large-volume enema solutions. Applied Nursing Research **2004**; 17(4): 265-74.

62. Fuchs EJ, Lee LA, Torbenson MS, et al. Hyperosmolar Sexual Lubricant Causes Epithelial Damage in the Distal Colon: Potential Implication for HIV Transmission. Journal of Infectious Diseases **2007**; 195(5): 703-10.

63. de Vries HJC, van der Bij AK, Fennema JSA, et al. Lymphogranuloma Venereum Proctitis in Men Who Have Sex With Men Is Associated With Anal Enema Use and High-Risk Behavior. Sexually Transmitted Diseases **2008**; 35(2): 203-8

64. Schreeder MT, Thompson SE, Hadler SC, et al. Hepatitis B in Homosexual Men: Prevalence of Infection and Factors Related to Transmission. Journal of Infectious Diseases **1982**; 146(1): 7-15.

65. Begay O, Jean-Pierre N, Abraham CJ, et al. Identification of personal lubricants that can cause rectal epithelial cell damage and enhance HIV type 1 replication in vitro. AIDS research and human retroviruses **2011**; 27(9): 1019-24.

66. Dezzutti CS, Brown ER, Moncla B, et al. Is Wetter Better? An Evaluation of Over-the-Counter Personal Lubricants for Safety and Anti-HIV-1 Activity. PloS one **2012**; 7(11): e48328.

67. Dezzutti CS, Rohan LC, Wang L, et al. Reformulated tenofovir gel for use as a dual compartment microbicide. Journal of Antimicrobial Chemotherapy **2012**; 67(9): 2139-42.

68. Gorbach PM, Weiss RE, Fuchs E, et al. The slippery slope: lubricant use and rectal sexually transmitted infections: a newly identified risk. Sex Transm Dis **2012**; 39(1): 59-64.

69. Vishwanathan SA, Morris MR, Wolitski RJ, et al. Rectal Application of a Highly Osmolar Personal Lubricant in a Macaque Model Induces Acute Cytotoxicity but Does Not Increase Risk of SHIV Infection. PloS one **2015**; 10(4): e0120021.

70. Amsden GW, Nafziger AN, Foulds G. Pharmacokinetics in serum and leukocyte exposures of oral azithromycin, 1,500 milligrams, given over a 3- or 5-day period in healthy subjects. Antimicrobial Agents & Chemotherapy **1999**; 43(1): 163-5.

71. Rapp RP. Pharmacokinetics and Pharmacodynamics of Intravenous and Oral Azithromycin: Enhanced Tissue Activity and Minimal Drug Interactions. Annals of Pharmacotherapy **1998**; 32(7-8): 785-93.

72. Nicolau DP. Predicting antibacterial response from pharmacodynamic and pharmacokinetic profiles. Infection **2001**; 29 Suppl 2: 11-5.

73. Romano J, Kashuba A, Becker S, Cummins J, Turpin J, Veronese oBotAPiHPTTP, Fulvia. Pharmacokinetics and pharmacodynamics in HIV prevention; current status and future directions: a summary of the DAIDS and BMGF sponsored think tank on pharmacokinetics (PK)/pharmacodynamics (PD) in HIV prevention. AIDS research and human retroviruses **2013**; 29(11): 1418-27.

74. Muto C, Liu P, Chiba K, Suwa T. Pharmacokinetic-pharmacodynamic analysis of azithromycin extended release in Japanese patients with common respiratory tract infectious disease. Journal of Antimicrobial Chemotherapy **2011**; 66(1): 165-74.

75. Craig WA. Pharmacokinetic/Pharmacodynamic Parameters: Rationale for Antibacterial Dosing of Mice and Men. Clinical Infectious Diseases **1998**; 26(1): 1-12.

76. Van Bambeke F, Tulkens PM. Macrolides: pharmacokinetics and pharmacodynamics. International journal of antimicrobial agents **2001**; 18, Supplement 1(0): 17-23.

77. Wang SA, Papp JR, Stamm WE, Peeling RW, Martin DH, Holmes KK. Evaluation of antimicrobial resistance and treatment failures for Chlamydia trachomatis: a meeting report. Journal of Infectious Diseases **2005**; 191(6): 917-23.

78. Welsh LE, Gaydos CA, Quinn TC. In vitro evaluation of activities of azithromycin, erythromycin, and tetracycline against Chlamydia trachomatis and Chlamydia pneumoniae. Antimicrob Agents Chemother **1992**; 36(2): 291-4.

79. Kintner J, Lajoie D, Hall J, Whittimore J, Schoborg RV. Commonly prescribed β-lactam antibiotics induce C. trachomatis persistence/stress in culture at physiologically relevant concentrations. Frontiers in Cellular and Infection Microbiology **2014**; 4: 44.

80. Boonleang J, Panrat K, Tantana C, Krittathanmakul S, Jintapakorn W. Bioavailability and pharmacokinetic comparison between generic and branded azithromycin capsule: A randomized, double-blind, 2-way crossover in healthy male thai volunteers. Clinical therapeutics **2007**; 29(4): 703-10.

81. Cooper MA, Nye K, Andrews JM, Wise R. The pharmacokinetics and inflammatory fluid penetration of orally administered azithromycin. Journal of Antimicrobial Chemotherapy **1990**; 26(4): 533-8.

82. Coates P, Daniel R, Houston AC, Antrobus JHL, Taylor T. An open study to compare the pharmacokinetics, safety and tolerability of a multiple-dose regimen of azithromycin in young and elderly volunteers. Eur J Clin Microbiol Infect Dis **1991**; 10(10): 850-2.

83. Dunn CJ, Barradell LB. Azithromycin. A review of its pharmacological properties and use as 3-day therapy in respiratory tract infections. Drugs **1996**; 51(3): 483-505.

84. Lucchi M, Damle B, Fang A, et al. Pharmacokinetics of azithromycin in serum, bronchial washings, alveolar macrophages and lung tissue following a single oral dose of extended or immediate release formulations of azithromycin. Journal of Antimicrobial Chemotherapy **2008**; 61(4): 884-91.

85. Liu P, Fang AF, LaBadie RR, Crownover PH, Arguedas AG. Comparison of Azithromycin Pharmacokinetics following Single Oral Doses of Extended-Release and Immediate-Release Formulations in Children with Acute Otitis Media. Antimicrobial Agents And Chemotherapy **2011**; 55(11): 5022-6.

86. Baschiera F, Fornai M, Lazzeri G, Blandizzi C, Bruschini P, Del Tacca M. Improved tonsillar disposition of azithromycin following a 3-day oral treatment with 20 mg kg-1 in paediatric patients. Pharmacol Res **2002**; 46(1): 95-100.

87. Curatolo W, Liu P, Johnson B, et al. Effects of Food on a Gastrically Degraded Drug: Azithromycin Fast-Dissolving Gelatin Capsules and HPMC Capsules. Pharm Res **2011**; 28(7): 1531-9.

88. Foulds G, Madsen P, Cox C, Shepard R, Johnson R. Concentration of azithromycin in human prostatic tissue. Eur J Clin Microbiol Infect Dis **1991**; 10(10): 868-71.

89. Baldwin D, Wise R, Andrews J, Ashby J, Honeybourne D. Azithromycin concentrations at the sites of pulmonary infection. European Respiratory Journal **1990**; 3(8): 886-90.

90. Krohn K. Gynecological tissue-levels of azithromycin. European Journal of Clinical Microbiology & Infectious Diseases **1991**; 10(10): 864-8.

91. Amsden GW. Erythromycin, clarithromycin, and azithromycin: are the differences real? Clin Ther **1996**; 18(1): 56-72; discussion 55.

92. Mardirossian G, Tagesson M, Blanco P, et al. A New Rectal Model for Dosimetry Applications. Journal of Nuclear Medicine **1999**; 40(9): 1524-31.

93. Padwal RS, Ben-Eltriki M, Wang X, et al. Effect of gastric bypass surgery on azithromycin oral bioavailability. Journal of Antimicrobial Chemotherapy **2012**; 67(9): 2203-6.

94. Idkaidek N, Arafat T. Saliva versus plasma pharmacokinetics: Theory and application of a salivary excretion classification system. Molecular Pharmaceutics **2012**; 9(8): 2358-63.

95. Vodstrcil L, Rupasinghe T, Tull D, et al. Post-treatment detection of azithromycin in high-vaginal swabs using liquid chromatography and tandem mass spectrometry (LC-MS/MS). In: 13th International Symposium on Human Chlamydial Infections. Pacific Grove California, 2013.

96. Ernst EJ, Klepser ME, Klepser TB, Nightingale CH, Hunsicker LG. Comparison of the serum and intracellular pharmacokinetics of azithromycin in healthy and diabetic volunteers. Pharmacotherapy **2000**; 20(6 I): 657-61.

97. Margaritis VK, Ismailos GS, Naxakis SS, Mastronikolis NS, Goumas PD. Sinus fluid penetration of oral clarithromycin and azithromycin in patients with acute rhinosinusitis. American Journal of Rhinology **2007**; 21(5): 574-8.

98. Guy R, Wand H, Franklin N, et al. Re-testing for chlamydia at sexual health services in Australia, 2004-08. Sex Health **2011**; 8(2): 242-7.

99. Matzneller P, Krasniqi S, Kinzig M, et al. Blood, Tissue, and Intracellular Concentrations of Azithromycin during and after End of Therapy. Antimicrobial Agents And Chemotherapy **2013**; 57(4): 1736-42.

100. Blandizzi C, Malizia T, Lupetti A, et al. Periodontal Tissue Disposition of Azithromycin in Patients Affected by Chronic Inflammatory Periodontal Diseases. Journal of Periodontology **1999**; 70(9): 960-6.

101. Amsden GW, Gray CL. Serum and WBC pharmacokinetics of 1500 mg of azithromycin when given either as a single dose or over a 3 day period in healthy volunteers. Journal of Antimicrobial Chemotherapy **2001**; 47(1): 61-6.

102. Liu P, Allaudeen H, Chandra R, et al. Comparative pharmacokinetics of azithromycin in serum and white blood cells of healthy subjects receiving a single-dose extended-release regimen versus a 3-day immediate-release regimen. Antimicrobial Agents & Chemotherapy **2007**; 51(1): 103-9.

103. Krichhoff RM, Laufen H, Schacke G, Kirchhoff G, Gallo E. Determination of azithromycin in gastric biopsy samples. Int J Clin Pharmacol Ther **1999**; 37(7): 361-4.

104. Ballow C, Amsden G, Highet V, Forrest A. Pharmacokinetics of Oral Azithromycin in Serum, Urine, Polymorphonuclear Leucocytes and Inflammatory vs Non-Inflammatory Skin Blisters in Healthy Volunteers. Clin Drug Investig **1998**; 15(2): 159-67.

105. Beringer P, Huynh KMT, Kriengkauykiat J, et al. Absolute Bioavailability and Intracellular Pharmacokinetics of Azithromycin in Patients with Cystic Fibrosis. Antimicrobial Agents And Chemotherapy **2005**; 49(12): 5013-7.

106. Harrison TS, Keam S. Azithromycin Extended Release. Drugs **2007**; 67(5): 773-92.

107. Di Paolo A, Barbara C, Chella A, Angeletti CA, Del Tacca M. Pharmacokinetics of azithromycin in lung tissue, bronchial washing, and plasma in patients given multiple oral doses of 500 and 1000 mg daily. Pharmacol Res **2002**; 46(6): 545-50.

108. Danesi R, Lupetti A, Barbara C, et al. Comparative distribution of azithromycin in lung tissue of patients given oral daily doses of 500 and 1000 mg. Journal of Antimicrobial Chemotherapy **2003**; 51(4): 939-45.

109. Chandra R, Liu P, Breen JD, et al. Clinical pharmacokinetics and gastrointestinal tolerability of a novel extended-release microsphere formulation of azithromycin. Clin Pharmacokinet **2007**; 46(3): 247-59.

110. Ehnhage A, Rautiainen M, Fang AF, Sanchez SP. Pharmacokinetics of azithromycin in serum and sinus fluid after administration of extended-release and immediate-release formulations in patients with acute bacterial sinusitis. International journal of antimicrobial agents **2008**; 31(6): 561-6.

111. Wilms EB, Touw DJ, Heijerman HGM. Pharmacokinetics of azithromycin in plasma, nlood, polymorphonuclear neutrophils and sputum during long-term therapy in patients with cystic fibrosis. Therapeutic drug monitoring **2006**; 28(2): 219-25.

112. Blandizzi C, Malizia T, Gherardi G, et al. Gastric mucosal distribution and clinical efficacy of azithromycin in patients with Helicobacter pylori related gastritis. Journal of Antimicrobial Chemotherapy **1998**; 42(1): 75-82.

113. Pfizer Australia. Zithromax product information. Australia, **2008**.

114. Pfizer USA. Zmax (azithromycin extended release) for oral suspension. **2012**.

115. Foulds G, Shepard RM, Johnson RB. The pharmacokinetics of azithromycin in human serum and tissues. J Antimicrob Chemother **1990**; 25: 73-82.

116. Shepard RM, Falkner FC. Pharmacokinetics of azithromycin in rats and dogs. J Antimicrob Chemother **1990**; 25 Suppl A: 49-60.

117. National Management Guidelines for Sexually Transmissible Infections Sexual Health Society of Victoria, **2008**.

118. Lazaro N. Sexually Transmitted Infections in Primary Care 2013 (RCGP/BASHH). 2nd ed. London: RCGP/BASHH, **2013**.

119. Centers for Disease Control and Prevention. Sexually transmitted diseases treatment guidelines, 2010 12/17/2010 Dec 17.

120. Lau C-Y, Qureshi AK. Azithromycin Versus Doxycycline for Genital Chlamydial Infections. A Meta-Analysis of Randomised Clinical Trials. Sex Transm Dis **2002**; 29(9): 497-502.

121. Handsfield HH. Questioning azithromycin for chlamydial infection. Sex Transm Dis **2011**; 38(11): 1028 - 9.

122. Horner P. The case for further treatment studies of uncomplicated genital Chlamydia trachomatis infection. Sex Transm Dis **2006**; 82: 340-3.

123. Horner PJ. Azithromycin antimicrobial resistance and genital Chlamydia trachomatis infection: duration of therapy may be the key to improving efficacy. Sex Transm Dis **2012**; 88(3): 154-6.

124. Sandoz KM, Rockey DD. Antibiotic resistance in Chlamydiae. Future Microbiol **2010**; 5(9): 1427-42.

125. Wang SA, Papp JR, Stamm WE, Peeling RW, Martin DH, Holmes KK. Evaluation of antimicrobial resistance and treatment failures for Chlamydia trachomatis: A meeting report. J Infect Dis **2005**; 191(6): 917-23.

126. Golden MR, Whittington WL, Handsfield HH, et al. Effect of expedited treatment of sex partners on recurrent or persistent gonorrhoea or chlamydial infection. NEJM **2005**; 352: 676-85.

127. Batteiger BE, Tu W, Ofner S, et al. Repeated Chlamydia trachomatis genital infections in adolescent women. J Infect Dis **2010**; 201(1): 42-51.

128. Foulds G, Johnson RB. Selection of dose regimens of azithromycin. J Antimicrob Chemother **1993**; 31: 39-50.

129. Worm AM, Osterlind A. Azithromycin levels in cervical mucus and plasma after a single 1.0g oral dose for chlamydial cervicitis. Gen Med **1995**; 71: 244-6.

130. Nirogi RVS, Kandikere VN, Shukla M, et al. Sensitive and selective liquid chromatography-tandem mass spectrometry method for the quantification of azithromycin in human plasma. Analytica Chimica Acta **2005**; 553: 1-8.

131. Chen BM, Liang YZ, Chen X, Liu SG, Deng FL, Zhou P. Quantitative determination of azithromycin in human plasma by liquid chromatography-mass spectrometry and its application in a bioequivalence study. Journal of pharmaceutical and biomedical analysis **2006**; 42(4): 480-7.

132. Yuzuak N, Ozden T, Eren S, Toptan S. Analysis of Azithromycin in Human Plasma by LC-MS-MS. Chromatographia **2007**; 66(1): S115-8.

133. Suchland RJ, Geisler WM, Stamm WE. Methodologies and cell lines used for antimicrobial susceptibility testing of Chlamydia spp. Antimicrob Agents Chemother **2003**; 47(2): 636-42.

134. Hocking JS, Vodstrcil L, Huston WM, et al. A cohort study of Chlamydia trachomatis treatment failure in women: a study protocol. BMC Infect Dis **2013**; 13(1): 379.

1. Water based, but less so silicone based lubricants, have been found to damage rectal mucosa

   <http://www.aidsmap.com/Study-finds-most-lubricants-damage-rectal-cells-and-some-increase-HIV-activity/page/1677777/> [↑](#footnote-ref-1)
2. Water based, but less so silicone based lubricants, have been found to damage rectal mucosa

   <http://www.aidsmap.com/Study-finds-most-lubricants-damage-rectal-cells-and-some-increase-HIV-activity/page/1677777/> [↑](#footnote-ref-2)
3. MIC was defined as the lowest drug concentration required for the complete inhibition of IFU of C. trachomatis compared with the controls. [↑](#footnote-ref-3)
4. Drug concentration in which 90% of inclusions appear aberrant [↑](#footnote-ref-4)
5. Amount of antimicrobial that prevents inclusion formation after antimicrobial removal and continued culture for 1 passage. [↑](#footnote-ref-5)
6. **500mg BD then 500mg OD 5/7 (total 3.5g)**: [AUC 0-12h] 1.77 to 3.18 mg.h/L, Cmax 0.41 to 0.62 mg/L

   250mg BD then 250mg OD 9/7 (total 2.75g): **day 1 vs day 10** : [AUC 0-12h (post 1^st^ dose) vs day 10 dose] 0.8 vs 1.22 mg.h/L, Cmax 0.2 vs 0.21 mg/L [↑](#footnote-ref-6)
